# Eicosapentaenoic Acid Improves Endothelial Nitric Oxide Bioavailability Via Changes in Protein Expression During Inflammation

**DOI:** 10.1161/JAHA.123.034076

**Published:** 2024-07-03

**Authors:** Samuel C. R. Sherratt, Peter Libby, Hazem Dawoud, Deepak L. Bhatt, R. Preston Mason

**Affiliations:** ^1^ Department of Molecular, Cellular, and Biomedical Sciences University of New Hampshire Durham NH USA; ^2^ Elucida Research Beverly MA USA; ^3^ Mount Sinai Fuster Heart Hospital Icahn School of Medicine at Mount Sinai New York NY USA; ^4^ Department of Medicine, Cardiovascular Division, Brigham and Women’s Hospital Harvard Medical School Boston MA USA; ^5^ Nanomedical Research Laboratory Ohio University Athens OH USA

**Keywords:** eicosapentaenoic acid, endothelial function, nitric oxide, proteomics, Endothelium/Vascular Type/Nitric Oxide, Inflammation, Proteomics, Basic Science Research, Growth Factors/Cytokines

## Abstract

**Background:**

Endothelial cell (EC) dysfunction involves reduced nitric oxide (NO) bioavailability due to NO synthase uncoupling linked to increased oxidation and reduced cofactor availability. Loss of endothelial function and NO bioavailability are associated with inflammation, including leukocyte activation. Eicosapentaenoic acid (EPA) administered as icosapent ethyl reduced cardiovascular events in REDUCE‐IT (Reduction of Cardiovascular Events With Icosapent Ethyl–Intervention Trial) in relation to on‐treatment EPA blood levels. The mechanisms of cardiovascular protection for EPA remain incompletely elucidated but likely involve direct effects on the endothelium.

**Methods and Results:**

In this study, human ECs were treated with EPA and challenged with the cytokine IL‐6 (interleukin‐6). Proinflammatory responses in the ECs were confirmed by ELISA capture of sICAM‐1 (soluble intercellular adhesion molecule‐1) and TNF‐α (tumor necrosis factor‐α). Global protein expression was determined using liquid chromatography–mass spectrometry tandem mass tag. Release kinetics of NO and peroxynitrite were monitored using porphyrinic nanosensors. IL‐6 challenge induced proinflammatory responses from the ECs as evidenced by increased release of sICAM‐1 and TNF‐α, which correlated with a loss of NO bioavailability. ECs pretreated with EPA modulated expression of 327 proteins by >1‐fold (*P*<0.05), compared with IL‐6 alone. EPA augmented expression of proteins involved in NO production, including heme oxygenase‐1 and dimethylarginine dimethylaminohydrolase‐1, and 34 proteins annotated as associated with neutrophil degranulation. EPA reversed the endothelial NO synthase uncoupling induced by IL‐6 as evidenced by an increased [NO]/[peroxynitrite] release ratio (*P*<0.05).

**Conclusions:**

These direct actions of EPA on EC functions during inflammation may contribute to its distinct cardiovascular benefits.

**Nonstandard Abbreviations and Acronyms**
EC: endothelial cell
eNOS: endothelial nitric oxide synthase
EPA: eicosapentaenoic acid
NO: nitric oxide
ONOO^−^
: peroxynitrite

**Clinical Perspective**

**What Is New?**

- Unlike other omega‐3 fatty acid formulations, eicosapentaenoic acid (EPA) administered as icosapent ethyl reduced cardiovascular events in high‐risk patients in relation to on‐treatment EPA blood levels through likely multiple mechanisms (REDUCE‐IT [Reduction of Cardiovascular Events With Icosapent Ethyl–Intervention Trial]).
- This study coupled endothelial protein expression to measurements of nitric oxide bioavailability to identify cellular pathways used by EPA in reversing endothelial dysfunction following a challenge by the cytokine IL‐6 (interleukin‐6).

**What Are the Clinical Implications?**

- The results showed that EPA reduced cytokine release and favorably modulated the expression of cytoprotective proteins associated with improved vasodilation as well as reduced oxidative stress and inflammation; these changes in endothelial function with EPA treatment would be expected to reduce the risk of atherothrombotic events in patients with high cardiovascular risk.

Endothelial‐derived NO (nitric oxide) regulates vascular tone, inhibits leukocyte adhesion and diapedesis, and interrupts platelet aggregation under physiological flow conditions.^1^ Loss of normal NO bioavailability accompanies atherogenesis and vascular injury.^2^ Endothelial NO synthase (eNOS), the enzyme responsible for NO generation, is a dimer composed of identical monomeric subunits.^3^ Regulation of eNOS involves a complex network of substrates and proteins, including BH_4_ (tetrahydrobiopterin), CAV1 (caveolin‐1), HMOX‐1 (heme oxygenase‐1), NADPH (nicotinamide adenine dinucleotide phosphate), reactive oxygen species (ROS), and asymmetric dimethylarginine (ADMA) among others.^1^, ^4^, ^5^ Additionally, membrane fatty acids regulate eNOS activity, as the omega‐3 fatty acids EPA (eicosapentaenoic acid) or DPA (docosapentaenoic acid) localized to caveolae can displace the eNOS inhibitor CAV1.^6^

Under normal conditions, eNOS catalyzes NO formation via coupling the oxidation of L‐arginine with reduction of molecular oxygen. However, under inflammatory conditions that increase oxidative stress (e.g., high glucose, smoking, hypertension), BH_4_ levels fail to maintain this redox reaction and lead to eNOS “uncoupling,”^7^ favoring production of superoxide ($O_{2}^{-}$).^7^ Such proinflammatory conditions can be induced by certain cytokines such as IL‐6 (interleukin‐6), which inhibits eNOS activation, participates in chronic inflammation, vascular dysfunction, and infectious agents.^8^, ^9^, ^10^ The O_2_^−^ produced by uncoupled eNOS can, in turn, react with cellular NO to form peroxynitrite (ONOO^−^) in a rapid, diffusion‐limited reaction. ONOO^−^ oxidizes BH_4_, leading to its further uncoupling.^11^ The ratio of [NO]/[ONOO^−^] release is therefore a sensitive indicator of eNOS coupling and cell function.^12^

Despite well‐controlled low‐density lipoprotein levels, residual cardiovascular risk persists for individuals with elevated triglycerides.^13^, ^14^ Efforts to reduce triglycerides with fibrates or niacin have thus far failed to reduce macrovascular cardiovascular risk on top of contemporary medical care.^15^ Treatment with IPE (icosapent ethyl), an ethyl ester form of EPA, reduces triglycerides in patients with dyslipidemia.^16^, ^17^ In REDUCE‐IT (Reduction of Cardiovascular Events With Icosapent Ethyl–Intervention Trial), patients on maximally‐tolerated statin therapy, with triglycerides ≥150 mg/dL and established cardiovascular disease or diabetes and at least 1 additional risk factor had a 25% relative risk reduction (*P*<0.0001) in a composite of cardiovascular end points when treated with IPE (4 g/d).^18^ There was no significant difference in ischemic event reduction across the baseline or achieved on‐treatment triglyceride levels, indicating a benefit with IPE independent of triglyceride lowering.^19^ Indeed, the biomarker with the strongest association with cardiovascular risk reduction was on‐treatment EPA levels,^20^ indicating that EPA itself mediates antiatherosclerotic benefits. Mechanistic insights accrued from the EVAPORATE (Effect of Vascepa on Improving Coronary Atherosclerosis in People With High Triglycerides Taking Statin Therapy) trial that used computed tomography angiography to demonstrate significant regression in low‐attenuation plaque volume and improvement in plaque morphology with 4 g/d IPE, as well as improvement in coronary flow.^21^, ^22^

EPA has various atheroprotective mechanisms, including improved endothelial function and reduced inflammation, lipid oxidation, leukocyte adhesion, and cholesterol crystal formation.^19^, ^23^, ^24^, ^25^, ^26^, ^27^, ^28^, ^29^, ^30^ In particular, EPA improved pulmonary endothelial function and eNOS coupling efficiency under conditions of inflammation caused by air pollution particulate matter.^31^ However, the effects of EPA on vascular EC function and protein expression following cytokine stimulation are unknown. This study tested the hypothesis that EPA modulates endothelial responses to IL‐6 to furnish new insight into the mechanisms of EPA‐induced cardiovascular event reduction.

## METHODS

The data that support the findings of this study are available from the corresponding author upon reasonable request.

### Materials

Primary HUVECs (human umbilical vein endothelial cells) were purchased from Lonza Inc. (Walkersville, MD), cultured in recommended complete endothelial cell growth medium and maintained at 37 °C in a 95% air/5% CO_2_ humidified incubator. Cell culture medium contained 2% fetal bovine serum, which facilitates the delivery of the fatty acids and elements required for IL‐6 signaling. EPA and IL‐6 were purchased from Sigma‐Aldrich (St Louis, MO). Recombinant human IL‐6 was animal‐component free, expressed in *Escherichia coli*, ≥98% (SDS‐PAGE), ≥98% (HPLC), free of endotoxin, and suitable for cell culture. The various fatty acids were solubilized in redistilled ethanol under a nitrogen atmosphere, and stock solutions were stored at −20 °C until use. Antibodies against HMOX‐1 were purchased from Cell Signaling (Catalog #: 70081), for eNOS from BD Biosciences (Catalog #: 610297), for Nrf2 (nuclear factor erythroid 2‐related factor 2) from Cell Signaling Technology (Catalog #: 12721), and for β‐actin from Santa Cruz Biotechnology (Catalog #: sc‐47 778).

### Proteomic Analysis

Relative protein production among the various treatments was measured using liquid chromatography‐mass spectrometry proteomic techniques as previously described.^31^ Confluent HUVECs were treated with 10 μmol/L EPA or equivolume vehicle for 2 hours in media containing 2% fetal bovine serum. This concentration of EPA was chosen based on achieved serum EPA levels in REDUCE‐IT and pharmacokinetic studies in which 4 g/d IPE led to the equivalent of low micromolar levels of EPA as used in this study.^32^ Cells were then challenged with IL‐6 (12 ng/mL) for 24 hours. After 24 hours, cells were pelleted and prepared for proteomic analysis.

Following methanol/chloroform extraction of the cell pellets, proteins were isolated and denatured, reduced, alkylated, and digested with trypsin. The resulting peptides from each sample replicate were then labeled by tandem mass tag 16plex labeling. Total protein levels in each sample were determined by a bicinchoninic acid assay, at which point the samples were combined into larger multiplexed pools.

Each multiplexed sample was then subjected to high pH reverse phase fractionation to increase the overall protein coverage and analyzed by liquid chromatography‐mass spectrometry using a Dionex UltiMate 3000 RSLC in tandem with a Q‐Exactive/Lumos Orbitrap Mass Spectrometer. The chromatography used a 3‐hour gradient on a Thermo Pepmap C18 column (100 Å pore size, 3.0 μm particle size, 75 μmol/L×150 mm) set at 50 °C. Mobile phase A was water with 0.1% formic acid, and mobile phase B was acetonitrile with 0.1% formic acid. The proteomic analysis was repeated at 40 μmol/L EPA to confirm the relative expression changes of key proteins identified at 10 μmol/L EPA treatment, and only proteins with the same direction of modulation and a *P* value <0.05 with both concentrations of EPA are discussed.

### Inflammatory Marker Analysis

To confirm proinflammatory actions of IL‐6 (at 12 ng/mL) in these ECs, we analyzed levels of sICAM‐1 (soluble intercellular adhesion molecule‐1) and TNF‐α (tumor necrosis factor‐α) in cell culture supernatant. Cells were incubated with IL‐6 or equivolume vehicle for 24 hours, at which point the supernatants were collected and levels of sICAM‐1 and TNF‐α were measured using commercially available ELISAs per the manufacturer's protocols (for sICAM‐1 we used catalog #BMS201 from Invitrogen (Frederick, MD, USA) and for TNF‐α we used catalog #BMS223‐4 from Invitrogen (Frederick, MD, USA)).

### Western Blot Analysis

Following proteomic analysis, expression of HMOX‐1, eNOS, Nrf2, and β‐actin (for normalization purposes) were evaluated by Western blot. Aliquots of cell lysate containing 7 μg protein were loaded into SDS‐PAGE gels and run at 100 V for 2 to 3 hours. Proteins were then transferred to nitrocellulose membranes and incubated with primary, then secondary antibodies, and intensity values were captured using chemiluminescence imager. Expression of HMOX‐1, Nrf2, and eNOS was normalized to β‐actin.

### Analysis of Mediators of Endothelial Function

Tandem electrochemical nanosensors simultaneously measured NO, ONOO^−^, and the [NO]/[ONOO^−^] ratio. Confluent ECs were first treated with EPA (10 μmol/L) or equivolume vehicle for 2 hours in 2% fetal bovine serum and then challenged with IL‐6 (12 ng/mL) for 24 hours. Cells were then rinsed with endothelial basal medium (EBM, Lonza, Inc.). Finally, eNOS activity was stimulated with 1.0 μmol/L of the calcium ionophore A23187 and the cells were analyzed for NO and ONOO^−^. The methods for performing and calibrating this measurement have been previously described and applied to our studies of these fatty acids and endothelial function.^28^, ^33^, ^34^

### Fatty Acid Level Analysis

Total cellular fatty acid levels were determined using gas chromatography. We limited the range of analytes to C14 to C24 with varying degrees of unsaturation. HUVECs were exposed to IL‐6 for 2 hours and then treated with EPA (40 μmol/L) or equivolume vehicle for 24 hours. Cells were then lysed in ice‐cold RIPA lysis buffer with protease inhibitor. An aliquot of the lysate was removed for protein content analysis by a bicinchoninic acid assay (ThermoPierce BCA Protein Assay Kit), which was subsequently used for normalization purposes. Total cellular fatty acids were extracted from lysed cells and derivatized to fatty acid methyl esters using methanol with 14% boron trifluoride. Fatty acid content was determined using gas chromatography via a Shimadzu GC‐2010 Gas Chromatograph with a Supelco SP‐2560, 100‐m fused silica capillary column (0.25 mm internal diameter, 0.2 μm film thickness). Sample spectra were compared with spectra of the fatty acid standards for each of the 24 fatty acid species analyzed. Fatty acid content normalized to the amount of protein per sample (mg/g total protein).

### Statistical Analysis

Raw mass spectral intensity data were analyzed using the bioinformatic package Differential Enrichment of Proteomic data version 1.4.1.^35^ Proteins of interest were identified as those in which there was a >1.0 fold change between treatment groups and *P* value <0.05. Gene Set Enrichment Analysis was also performed on proteins of interest. For pathway analysis, significantly modulated pathways were those with an adjusted *P* value <0.05, which adjusts for the size of a given gene set and for multiple hypothesis testing. Data analysis was performed using Thermo Xcalibur Qualbrowser, Proteome Discoverer 2.2, PEAKS Studio X+. Gene sets were also compared within treatment groups and ranked according to adjusted *P* value and gene ratio using edgeR.^36^, ^37^ The *P* value calculations for the pathways found in the Gene Ontology database were based on previously published methods.^38^

For the endothelial function measurements of NO and ONOO^−^ release, data were presented as the mean±SEM for of treatment groups (N=4–5). Western blot intensity values were presented as the mean±SD for N=3 replicates in each treatment group. Levels of sICAM‐1 and TNF‐α were presented as the mean±SD for N=3–6 replicates in each treatment group. Differences between groups were analyzed using ANOVA followed by Tukey–Kramer multiple comparisons post hoc analysis (for comparisons among 3 or more groups) or unpaired, 2‐tailed Student's *t* test for comparisons between 2 groups. Alpha error was set to 0.05 in this study. Institutional review board approval was not required for this study.

## RESULTS

### Inflammatory Effects of IL‐6

We first sought to confirm the proinflammatory effects of IL‐6 at 12 ng/mL in HUVECs. Following 24‐hour incubation, IL‐6 treatment significantly increased levels of sICAM‐1 (83%, *P*<0.0001) and TNF‐α (83%, *P*<0.01) in the cell culture supernatant compared with vehicle‐treated controls. These data are presented in Figure S1. Having established the proinflammatory response of IL‐6 in these cells, we proceeded to characterize global protein changes in the absence or presence of EPA.

### Cellular Proteomic Analysis: Endothelial Function

**Figure 1:**
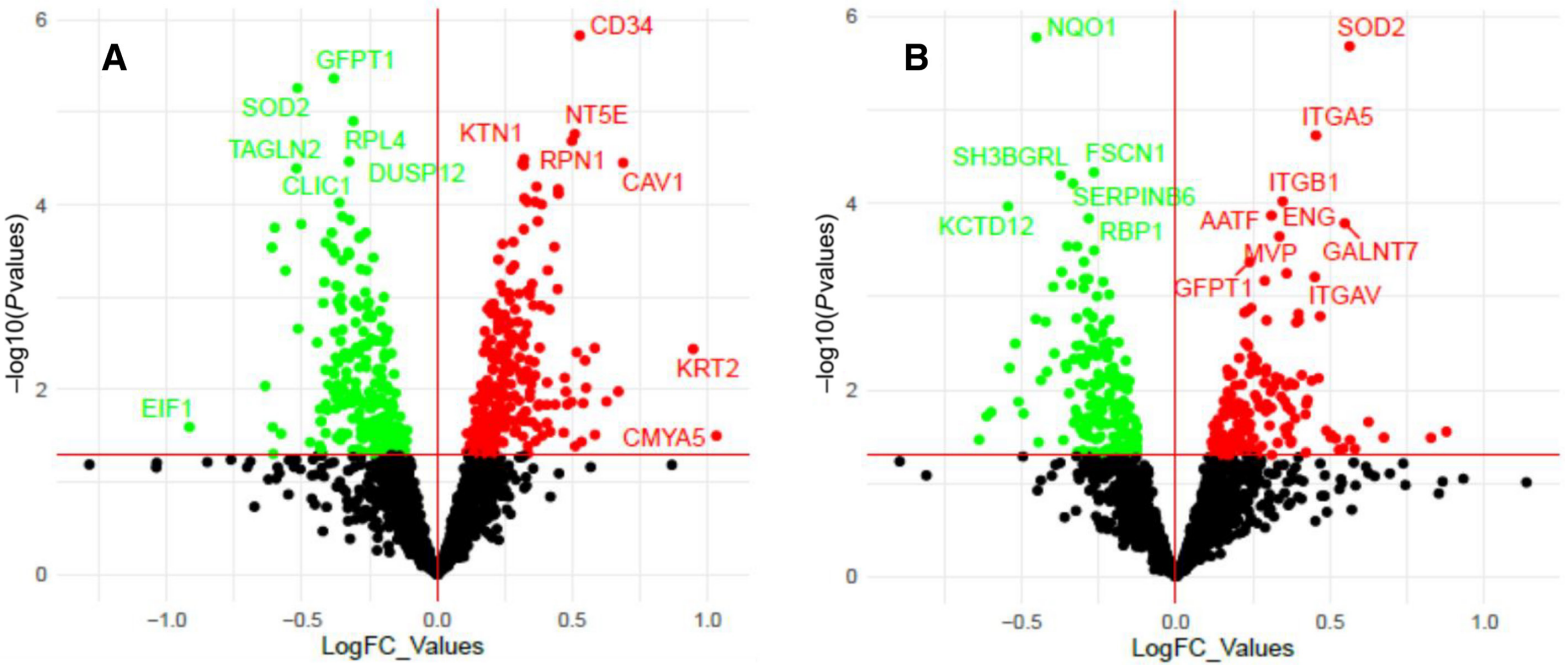
Volcano plot of protein expression changes in (A) IL‐6 alone vs vehicle, and (B) EPA+IL‐6 vs IL‐6 alone. Vertical lines indicate expression fold change >1.0 (up or downregulation). Horizontal line indicates *P* value of 0.05; thus, protein marks above the line correlate to *P*<0.05. Only proteins that fall into the upper left or upper right section of the graph are considered significant. EPA indicates eicosapentaenoic acid; FC, fold change; and IL‐6, interleukin‐6.

**Figure 2:**
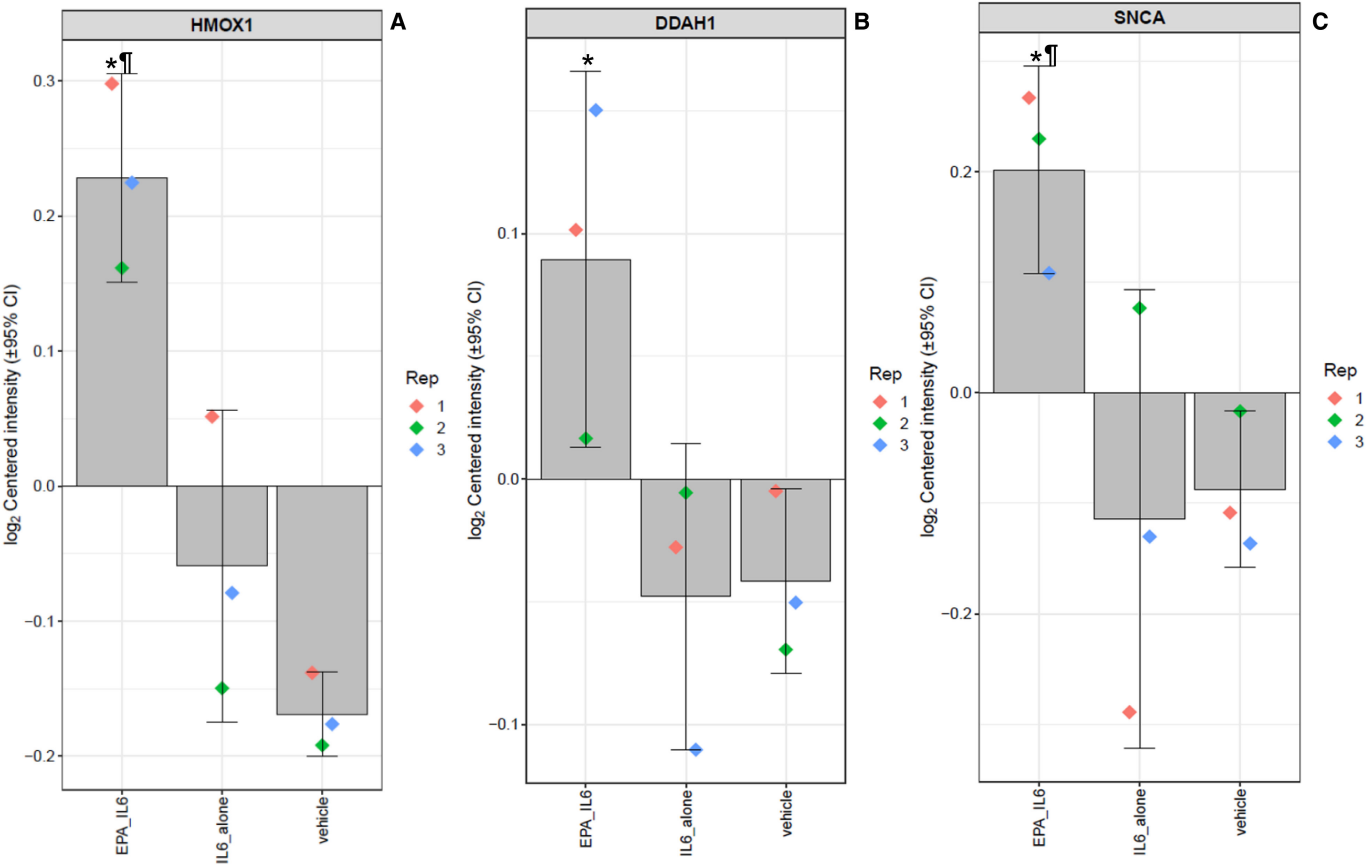
Relative expression levels of (A) heme oxygenase‐1, (B) dimethylarginine dimethylaminohydrolase‐1, and (C) α‐synuclein between treatments. The log2‐normalized intensity values of each protein among the treatment groups, with the value of each replicate shown by the colored diamonds, the average indicated by the gray bar, and the 95% CI shown by the error bars. Statistical indicators: *P<0.05 vs IL‐6 vs vehicle; ^¶^ *P*<0.05 vs vehicle. DDAH1 indicates dimethylarginine dimethylaminohydrolase‐1; EPA, eicosapentaenoic acid; HMOX1, heme oxygenase‐1; IL‐6, interleukin‐6; and SNCA, α‐synuclein.

**Figure 3:**
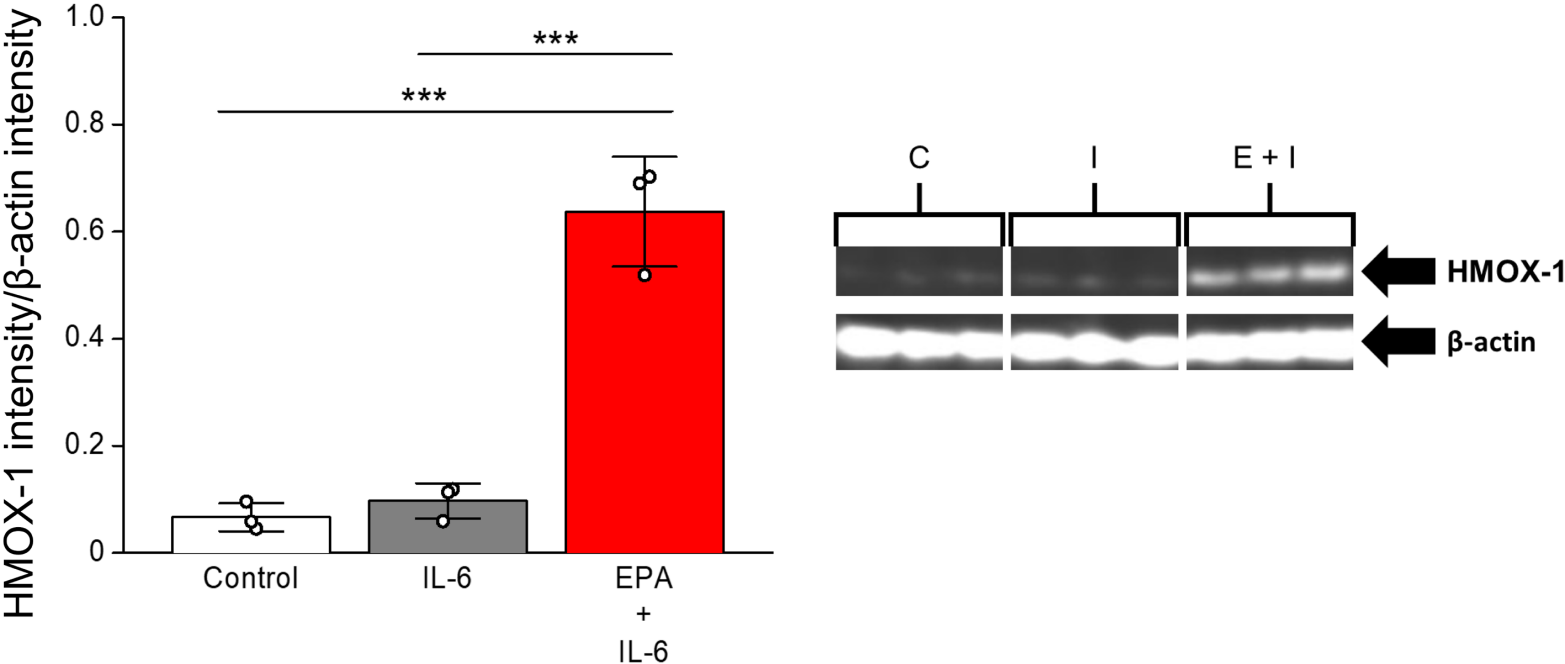
Expression of heme oxygenase‐1 in each treatment group by Western blot. The expression of HMOX‐1 was normalized to β‐Actin levels. Statistical indicators: ***P<0.001 (Tukey–Kramer multiple comparisons test; overall ANOVA: *P*<0.0001, *F*=75.330). Values are mean±SD (N=3). C, control; I, IL‐6; E+I, EPA+IL‐6 EPA indicates eicosapentaenoic acid; HMOX‐1, heme oxygenase‐1; and IL‐6, interleukin‐6.

Volcano plot summaries of global proteomic data are found in Figure 1. Both treatment comparisons yielded an even distribution of increased and decreased proteins. IL‐6 alone significantly increased 229 proteins and decreased 244 proteins compared with vehicle control, whereas EPA treatment before IL‐6 challenge increased 176 proteins and decreased 166 proteins relative to IL‐6 challenge alone. Several proteins stood out given their known role in cytoprotection and endothelial function. The log2‐transformed raw mass spectral intensity data from each treatment replicate for these proteins are summarized in Figure 2A through 2C. EPA pretreatment increased expression of the cytoprotective protein HMOX‐1 by 1.2‐fold relative to IL‐6 challenge alone (*P*=0.002). There was also a significant increase in expression of DDAH‐1 (dimethylarginine dimethylaminohydrolase‐1, 1.1‐fold, *P*=0.046). This protein plays a role in increasing NO production by breaking down endogenous inhibitors of eNOS such as ADMA and monomethyl arginine in the vasculature.^39^ Interestingly, there was no effect on total eNOS expression with EPA treatment. Finally, there was a 1.2‐fold increase in SNCA (α‐synuclein) with EPA relative to IL‐6. SNCA is both expressed within and secreted from ECs and can play a role in eNOS activation (discussed more later). EPA treatment at 40 μmol/L confirmed the increased relative expression of HMOX‐1 2.1‐fold, DDAH1 1.1‐fold, and SNCA 1.2‐fold compared with IL‐6 alone (all *P*<0.001) with no change in eNOS (Figure S2 and Table S1). The effects of EPA on HMOX‐1 and eNOS were further confirmed by Western blots (Figure 3, Figure S3): EPA increased HMOX‐1 557% versus IL‐6 alone (0.64±0.10 versus 0.10±0.03, intensity normalized to β‐actin), yet had no significant effect on eNOS.

### Cellular Proteomic Analysis: Oxidative Stress Response

**Figure 4:**
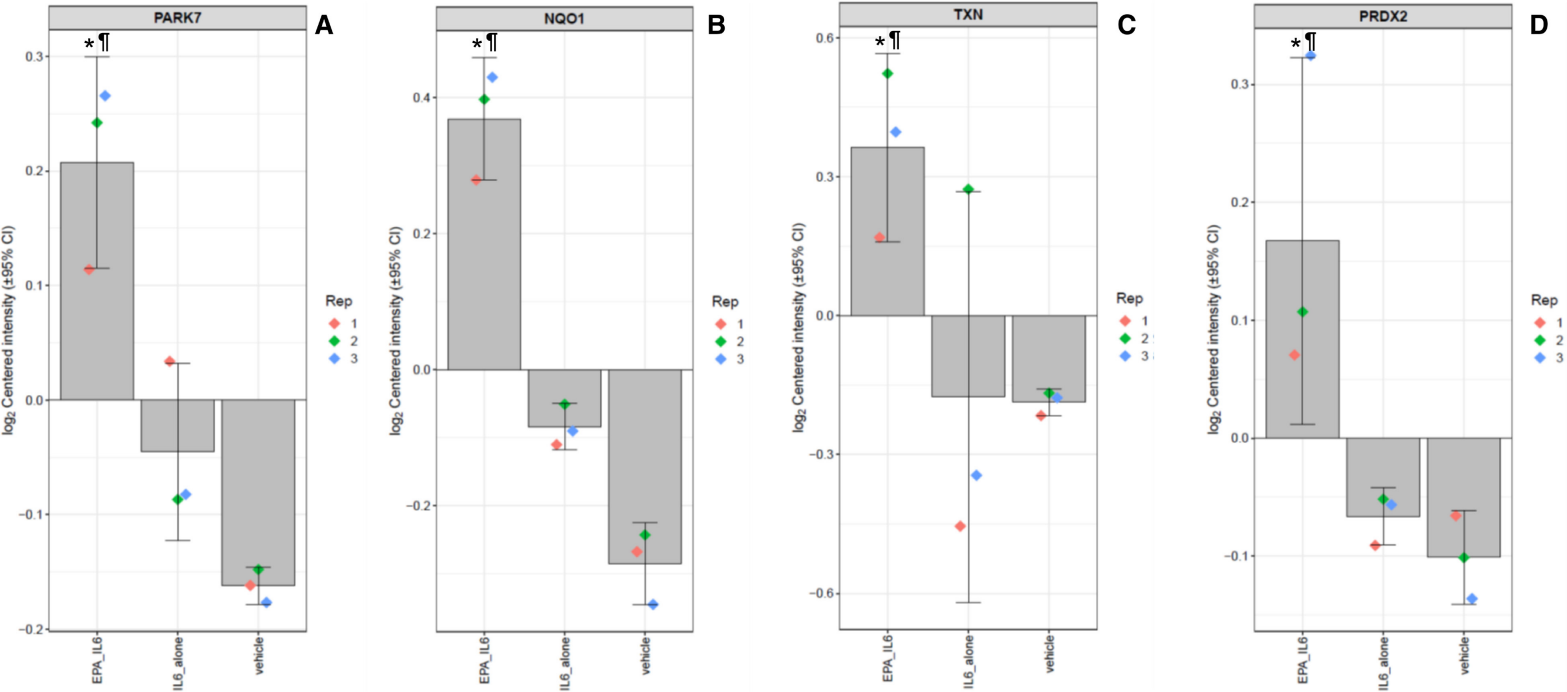
Relative expression levels of (A) Parkinson disease protein 7, (B) NAD(P)H quinone oxidoreductase‐1, (C) thioredoxin, and (D) peroxiredoxin‐2 between treatments. The log2‐normalized intensity values of each protein among the treatment groups, with the value of each replicate shown by the colored diamonds, the average indicated by the gray bar, and the 95% CI shown by the error bars. Statistical indicators: *P<0.05 vs IL‐6 vs Vehicle; ^¶^ *P*<0.05 vs vehicle. EPA indicates eicosapentaenoic acid; IL‐6, interleukin‐6; NQO1, NAD(P)H quinone oxidoreductase‐1; PARK7, Parkinson disease protein 7; PRDX2, peroxiredoxin‐2; and TXN, thioredoxin.

The Gene Set Enrichment Analysis revealed a total of 232 pathways were significantly modulated by EPA treatment relative to IL‐6. Among these pathways was the “cellular response to oxidative stress” (Gene Ontology:0034599), in which EPA affected 7 proteins (adjusted *P* value=0.020). The log2‐transformed raw mass spectral intensity data from each treatment replicate for several of these proteins are summarized in Figure 4A through 4D, including PRDX2 (peroxiredoxin‐2), TXN (thioredoxin), and PARK7 (Parkinson disease protein 7). TXN, and PRDX2 are both involved in detoxification of ROS, including hydrogen peroxide, and each was increased by EPA treatment relative to IL‐6 challenge by 1.5‐ and 1.2‐fold, respectively (all *P*<0.05). There was also a 1.2‐fold increase in PARK7 with EPA treatment (*P*=0.001). This protein stabilizes Nrf2, a transcription factor that regulates expression of antioxidant response elements.^40^ Among the proteins which Nrf2 transcriptionally regulates are HMOX‐1, TXN, and NQO1 (NAD(P)H quinone oxidoreductase‐1). EPA treatment increased these proteins, indicating that EPA may induce the increase in HMOX‐1, TXN, NQO1, and other antioxidant proteins via PARK7‐mediated stabilization of Nrf2. The relative increases in expression of PARK7 (1.1‐fold), TXN (1.2‐fold), NQO1 (1.3‐fold), and PRDX2 (1.1‐fold) were confirmed with EPA at 40 μmol/L (Figure S2 and Table S1). We measured Nrf2 expression via Western blot, and there was no change in overall Nrf2 expression (Figure S4) in accordance with previous results which found EPA increased translocation of Nrf2 to the nucleus whereas Nrf2 expression remained consistent.^41^

### Cellular Proteomic Analysis: Neutrophil Degranulation

**Figure 5:**
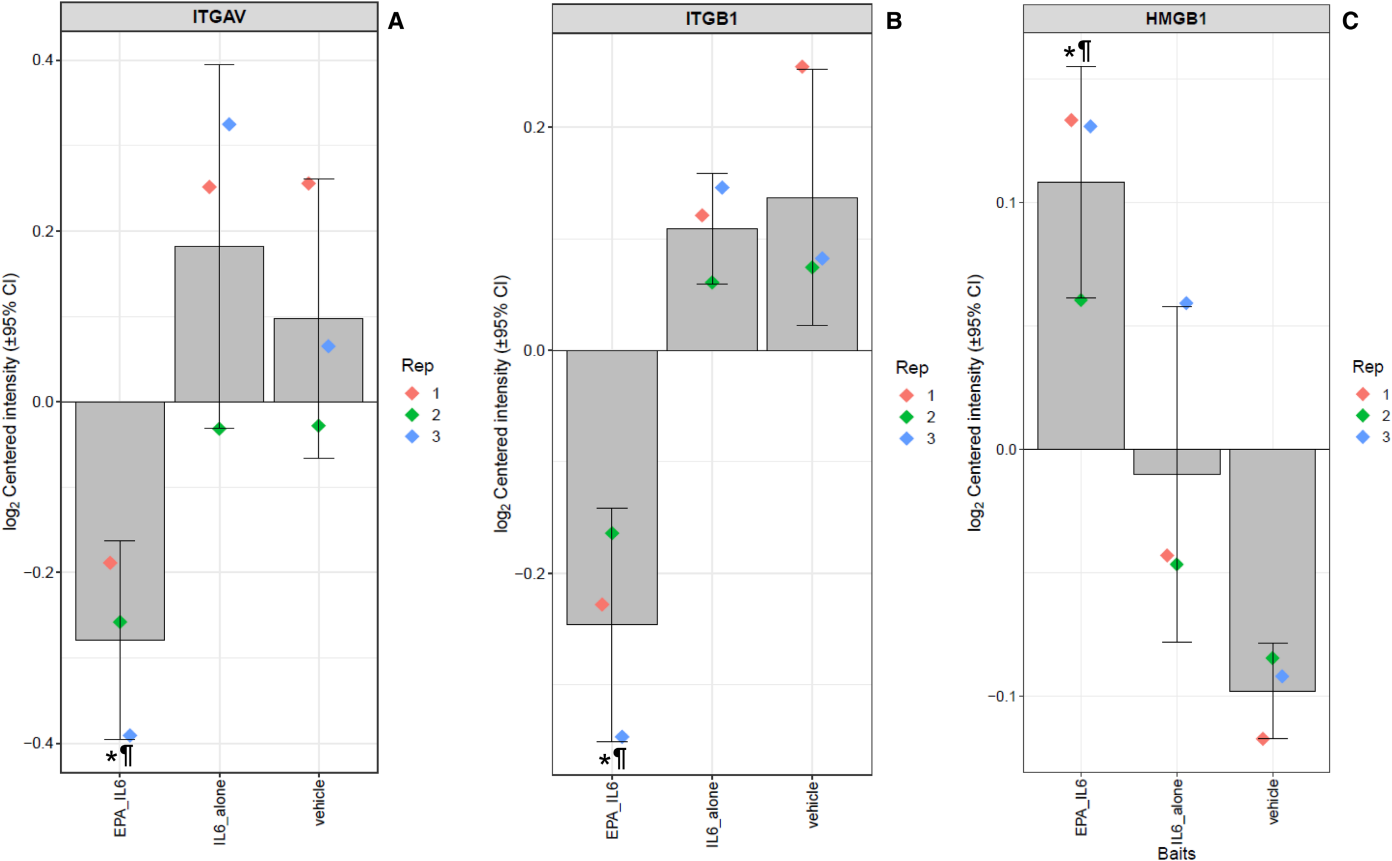
Relative expression levels of (A) integrin αV, (B) integrin β1, and (C) high mobility group box 1 between treatments. The log2‐normalized intensity values of each protein among the treatment groups, with the value of each replicate shown by the colored diamonds, the average indicated by the gray bar, and the 95% CI shown by the error bars. *P<0.05 vs IL‐6 vs vehicle; ^¶^ *P*<0.05 vs vehicle. EPA indicates eicosapentaenoic acid; HMGB1, high mobility group box 1; IL‐6, interleukin‐6; ITGAV, integrin αV; and ITGB1, integrin β1.

The complete list of significantly modulated pathways can be found in Table S2. Among the most significantly modulated pathways with EPA pretreatment, with regards to adjusted *P* value and total number of proteins modulated within the pathway, was the proinflammatory cluster denoted “neutrophil degranulation” (Gene Ontology:0043312), in which EPA modulated 32 proteins (pathway adjusted *P* value=4.01×10^−7^). This included decreases in expression of ITGAV (integrin αV) by 1.4‐fold (*P*=0.024), ITGB1 (integrin B1, 1.3‐fold, *P*=9.57×10^−5^), and increased expression of HMGB1 (high mobility group box 1, 1.1‐fold, *P*=0.025) (Figure 5). The relative changes in expression of ITGAV (1.1‐fold), ITGB1 (1.1‐fold), and HMGB1 (1.1‐fold) were confirmed with EPA at 40 μmol/L (all *P*<0.01) (Figure S2 and Table S1).

### Analysis of Mediators of Endothelial Function

**Figure 6:**
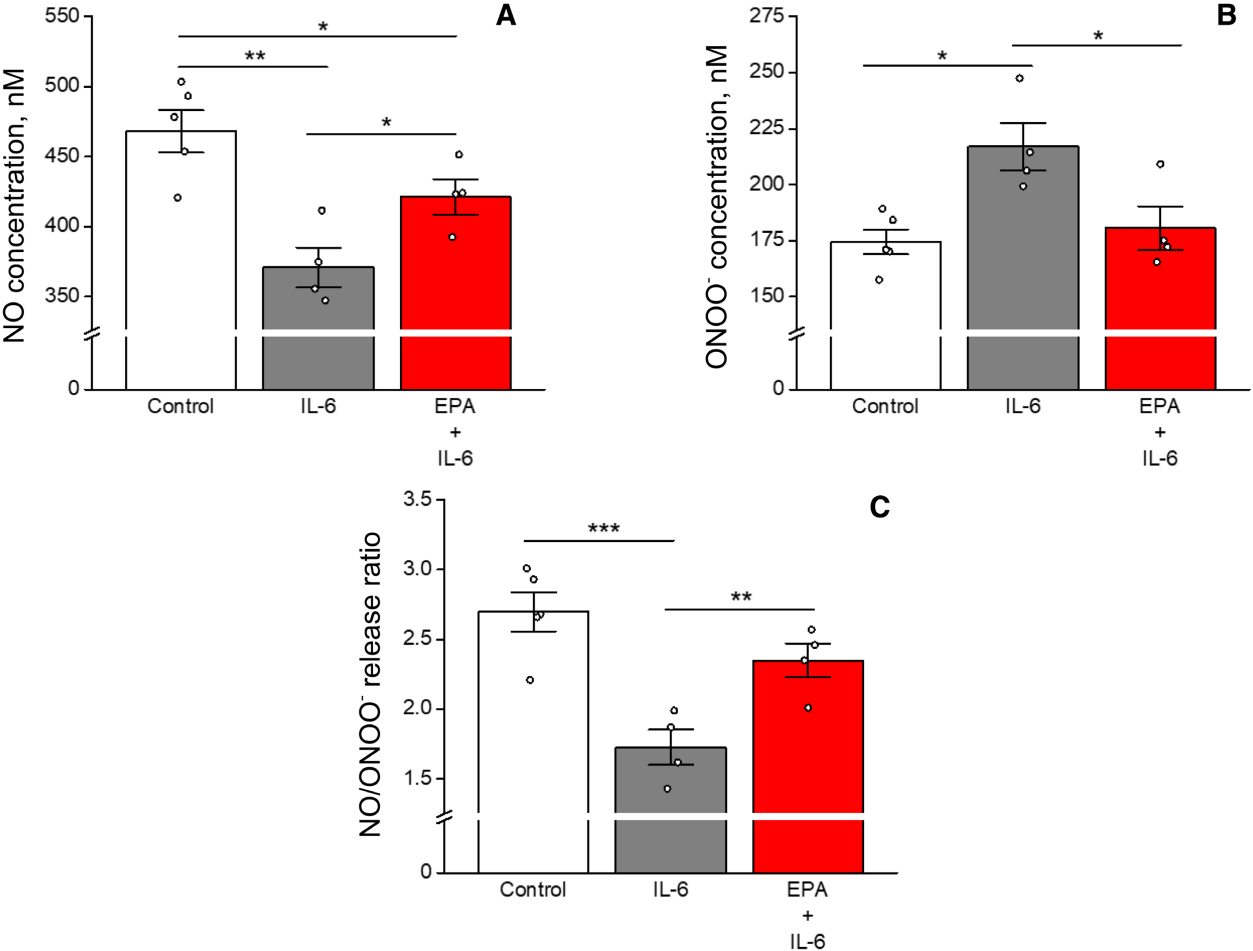
Effects of EPA on (A) nitric oxide release, (B) peroxynitrite release, and (C) the NO/ONOO− release ratio under inflammatory conditions with IL‐6. Statistical indicators: NO stats—**P<0.01; *P<0.05 (Tukey–Kramer multiple comparisons test; overall ANOVA: *P*=0.0024) *P=0.0378 vs IL‐6 alone (unpaired, 2‐tailed Student's *t* test; *t*=2.654, df=6). ONOO− stats—*P<0.05 (Tukey–Kramer multiple comparisons test; overall ANOVA: *P*=0.0119). Ratio stats—***P<0.001; **P<0.01 (Tukey–Kramer multiple comparisons test; overall ANOVA: *P*=0.0013). Values are mean±SEM (N=4–5). EPA indicates eicosapentaenoic acid; IL‐6, interleukin‐6; NO, nitric oxide; and ONOO−, peroxynitrite.

The results of the endothelial function analysis are summarized in Figure 6A through 6C. Treatment with IL‐6 reduced NO release from ECs by 21% compared with vehicle after 24 hours (371±14 versus 468±15 nmol/L, *P*<0.01). Pretreatment with EPA reversed this effect, causing a 13% increase in NO release compared with IL‐6 treatment (421±12 versus 371±14 nmol/L, *P*=0.0378). IL‐6 also caused a 24% increase in ONOO^−^ release versus vehicle (217±11 versus 174±6 nmol/L, *P*<0.05), and EPA produced a 17% decrease in ONOO^−^ release compared with IL‐6 (181±10 versus 217±11 nmol/L, *P*=0.0453) (Figure 6B). IL‐6 treatment further reduced the [NO]/[ONOO^−^] release ratio by 36% compared with vehicle‐treated cells (1.73±0.13 versus 2.70±0.14, *P*<0.001, Figure 6C). EPA reversed this effect by 36% compared with IL‐6 alone (2.35±0.12 versus 1.73±0.13, *P*<0.05).

### EPA Changes Cellular Fatty Acid Composition

The results of the fatty acid analysis are summarized in Table S3. IL‐6 treatment alone did not significantly change fatty acid levels. However, the changes in protein expression and endothelial function correlated with increased levels of EPA following EPA treatment. There were no changes in arachidonic acid (AA) levels by any treatment. This led to a large, significant increase in the EPA/AA ratio by EPA treatment (21‐fold, *P*<0.001). There was also a significant increase in the omega‐3 fatty acid DPA with EPA treatment (4‐fold, *P*<0.001). The biosynthesis of DPA occurs when EPA undergoes an elongation reaction by elongase 2 or 5. There was no EPA‐induced increase in DHA levels. These changes in fatty acids are consistent with previously published results in ECs.^29^

## DISCUSSION

**Figure 7:**
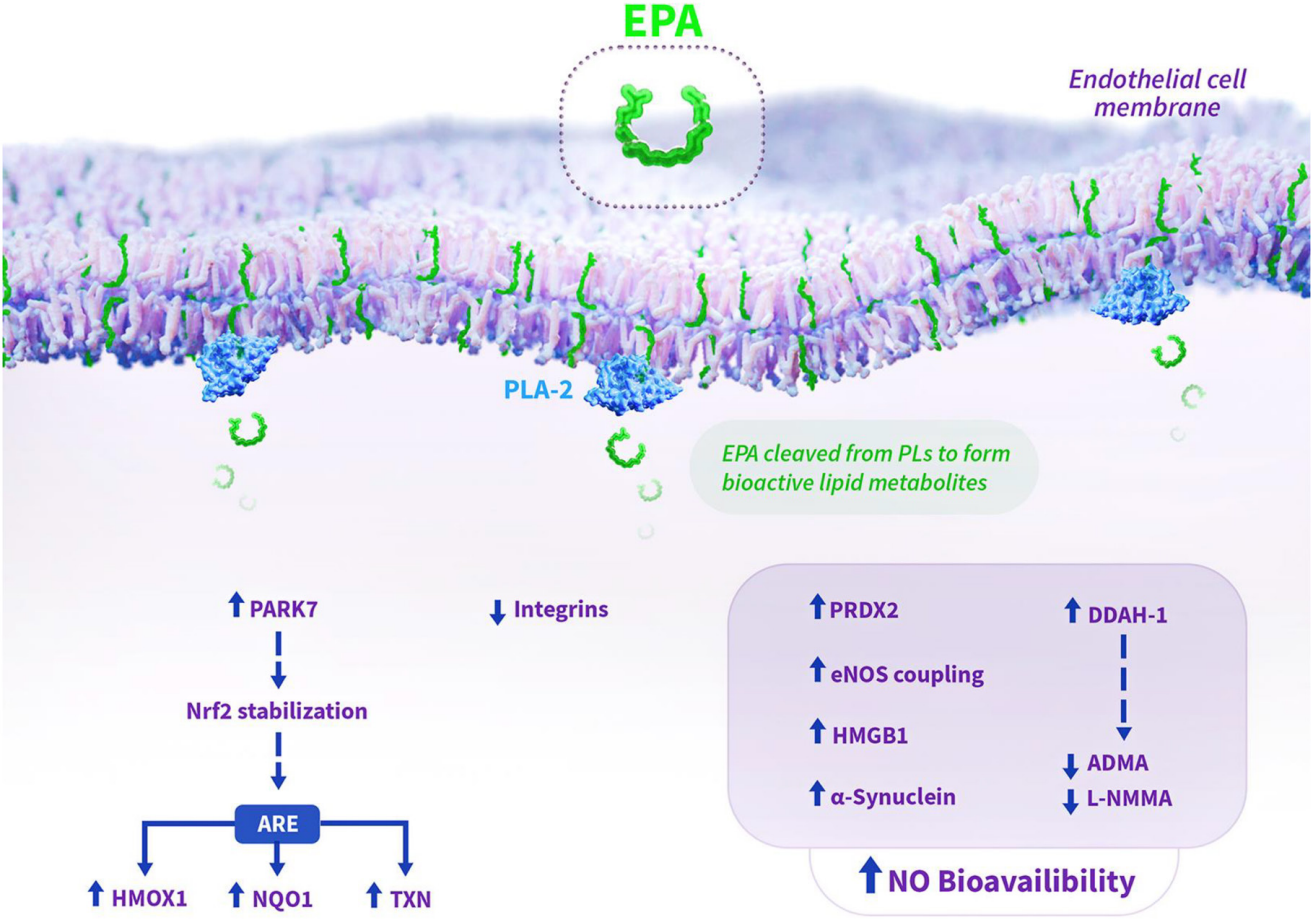
Summary of key findings from current study: EPA‐mediated endothelial protection during inflammation. EPA facilitates improved NO bioavailability and endothelial cell protection through various mechanisms elucidated in the current study. EPA is known to concentrate in phospholipids in the cell membrane, where it can be liberated by PLA‐2 to activate various intracellular actions. By improving eNOS coupling efficiency, attenuating oxidative stress, and modulating endogenous inhibitors (L‐NMMA, ADMA), EPA treatment abates endothelial dysfunction. Additionally, EPA increases expression of PARK7, which stabilizes Nrf2 to increase expression of genes within ARE in DNA, including HMOX‐1, TXN, and NQO1. Increased expression of α‐synuclein with EPA may also contribute to the improved NO bioavailability, as recombinant α‐synuclein has been shown to improve NO release. EPA‐mediated increased levels of HMGB1 may represent a novel mechanism for improved NO bioavailability, as intracellular HMGB1 has been shown to be required for normal vasomotor function and NO release. Solid arrows indicate observed changes with EPA treatment in the current study, while dashed arrows represent hypothesized effects based on changes in protein expression with EPA treatment. ADMA indicates asymmetric dimethylarginine; ARE, antioxidant response element; DDAH‐1, dimethylarginine dimethylaminohydrolase‐1; eNOS, endothelial nitric oxide synthase; EPA, eicosapentaenoic acid; HMGB1, high mobility group box protein 1; HMOX1, heme oxygenase‐1; IL‐6, interleukin‐6; L‐NMMA, N(G)‐monomethyl L‐arginine; NO, nitric oxide; NQO1, NAD(P)H quinone oxidoreductase‐1; Nrf2, nuclear factor erythroid 2‐related factor; PARK7, Parkinson's Disease protein 7; PLs, phospholipids; PLA‐2, phospholipase A2; PRDX2, peroxiredoxin‐2; and TXN, thioredoxin.

This study provides novel insight into the mechanisms by which EPA may influence atherothrombotic events. We summarize the key findings in Figure 7. We evaluated the effects of EPA on protein expression and NO bioavailability under conditions of inflammatory stimulation by IL‐6. Treatment with IL‐6 alone incited proinflammatory actions by ECs as evidenced by increased release of sICAM‐1 and TNF‐α. IL‐6 treatment also caused pronounced alterations in processes implicated in EC dysfunction as evidenced by reduced NO levels concomitant with increased ONOO^−^ release, leading to a decrease in the [NO]/[ONOO^−^] release ratio, indicating a highly unfavorable balance between protective versus injurious functions. This finding agrees with previous studies that IL‐6 induces oxidative stress and EC dysfunction through inhibition of eNOS and increased NADPH  oxidase activity.^42^

The ECs pretreated with EPA significantly reversed the [NO]/[ONOO^−^] release ratio compared with IL‐6 treatment alone without changes in eNOS levels. This improvement in NO bioavailability correlated with increased expression of proteins that indirectly modulate eNOS activity. One such protein was DDAH‐1, which catalyzes the breakdown of endogenous eNOS inhibitors such as ADMA and monomethyl arginine.^39^, ^43^ The increase in SNCA is another novel mechanism of eNOS activation as its knockout in animal models leads to an increase in systolic blood pressure and EC dysfunction.^44^ In HUVECs, silencing of SNCA decreases eNOS expression and phosphorylation, whereas exogenous delivery of recombinant SNCA increases NO release. Additionally, SNCA exhibits anti‐inflammatory actions in HUVECs challenged with TNF‐α as shown by a decrease in nuclear factor kappa B activity and VCAM‐1 (vascular cell adhesion protein 1) expression.^44^ These EPA‐induced increases in proteins that modulate eNOS may contribute to the increased NO bioavailability.

This study showed a large, significant increase in HMOX‐1 levels in ECs pretreated with EPA using multiple analytical techniques. HMOX‐1 expression is influenced by NO production.^45^ The primary enzymatic function of HMOX‐1 is the catalytic degradation of free heme to iron, biliverdin, and carbon monoxide. The degradation of heme, which is proinflammatory and cytotoxic when unbound, is a crucial cytoprotective mechanism of HMOX‐1.^45^, ^46^ HMOX‐1 provides protection to the vasculature through both NO‐dependent and ‐independent mechanisms.^47^, ^48^ The various antioxidant, anti‐inflammatory, and endothelial protective effects of HMOX‐1 may limit the progression of atherosclerosis as shown in models of disease.^49^, ^50^, ^51^

Multiple studies demonstrated that EPA induces HMOX‐1 expression in ECs under various inflammatory conditions, including cytokines, hydrogen peroxide, and air pollution.^31^, ^41^, ^52^ EPA treatment induced a translocation of the transcription factor Nrf2 to the nucleus, leading to an increase in transcription and translation of HMOX‐1. The *HMOX‐1* gene is one of multiple antioxidant and cytoprotective proteins contained within an antioxidant response element‐promoter region, which also includes *TXN* and *NQO1*.^40^, ^53^ In the present study, EPA treatment not only increased HMOX‐1 but also TXN and NQO1, which both have their own ROS detoxification effects.^53^, ^54^ Interestingly, there was a significant increase in PARK7 levels with EPA treatment known to stabilize Nrf2 and prevent it from binding to Keap1 (Kelch‐like ECH‐associated protein 1), which would otherwise lead to its enzymatic breakdown.^40^ Thus, EPA may not only induce translocation of Nrf2 to increase HMOX‐1, TXN, and NQO1 levels but also ensure Nrf2 remains intact by stimulating PARK7 expression. Together, these data imply that HMOX‐1 may facilitate certain endothelial and antiatherosclerotic benefits of EPA.

Another protein linked to both inflammatory responses and NO bioavailability is HMGB1, which was significantly increased by EPA at multiple concentrations. This chromatin‐associated protein can be posttranslationally modified under various cellular conditions, including increased levels of ROS, leading to its translocation from the nucleus to the cytosol.^55^ When secreted or passively released, HMGB1 acts as a proinflammatory signaling molecule. However, intracellular actions of HMBG1 in ECs have been associated with normal vasomotor function, oxidative stress response, and NO bioavailability whereas deletion and knockdown of HMGB1 leads to a decrease in eNOS phosphorylation, and NO release, increased blood pressure and ROS, and loss of endothelial‐dependent vasorelaxation.^56^ Interestingly, previous studies have shown crosstalk between Nrf2, HMOX‐1, and HMGB1. Increased expression of HMOX‐1 decreased HMGB1 translocation to the cytosol and subsequent secretion or release.^55^, ^57^

This axis has also been implicated in ischemia reperfusion injury in myocardial tissue. Increased levels of ONOO− induce HMGB1 release in models of cardiac and cerebral reperfusion injury, whereas scavenging of ONOO− decreases HMGB1 release.^58^, ^59^ The results from our current study support these results and suggest multiple mechanisms of HMGB1 retention within ECs. EPA both increased HMOX1 via PARK7 stabilization of Nrf2 and decreased ONOO−, leading to increased HMGB1 levels within the cells. This, in turn, facilitates the previously reported beneficial effects of HMGB1 on NO bioavailability.^56^ Further investigation into the effects of EPA‐induced expression of HMOX‐1 and HMGB1 retention in other systems, including those modeling ischemia reperfusion injury, may reveal other novel protective effects.

As mentioned previously, EPA significantly increased expression of numerous proteins involved in ROS detoxification, including PRDX2, NQO1, TXN, and PARK7. By increasing expression of these proteins, EPA may reduce oxidative stress in ECs, thereby sustaining EC function and NO bioavailability under inflammatory conditions as shown in this and previous studies.^6^, ^29^, ^31^ Indeed, ROS are generated from uncoupled eNOS and can damage necessary cofactors, such as tetrahydrobiopterin, required for proper eNOS function.^60^ Thus, activating ROS detoxification pathways directly affects NO bioavailability and normal vasomotor function. These effects may therefore contribute to the antiatherosclerotic activity of EPA observed in clinical trials. For example, PRDX2 can inhibit H_2_O_2_ production induced by cytokines in ECs ex vivo and slow atherosclerosis in animal models of disease.^61^, ^62^

We previously observed that EPA significantly improved NO bioavailability in human ECs challenged with oxidized small dense low‐density lipoprotein, an effect that was enhanced in combination with a statin.^28^ We also recently reported that EPA improves eNOS coupling efficiency following air pollution particulate matter injury in pulmonary endothelial cells.^31^ Additionally, EPA improves NO bioavailability in ECs in the absence of inflammation as compared with DHA or AA, and this effect correlated with specific changes in fatty acid composition, including increased levels of EPA, DPA, and a 10‐fold increase in the EPA/AA ratio without changes in DHA.^29^ The current results extend our understanding of the effects of EPA on EC function and NO metabolism under conditions of inflammation, including modulation of protein expression. We again observed a significant increase in levels of EPA, DPA, and the EPA/AA ratio with EPA treatment in these endothelial cells. The strongest biomarker found to be predictive of risk reduction in REDUCE‐IT was on‐treatment EPA level, suggesting that EPA itself conveys the antiatherosclerotic action rather than an EPA‐mediated change in dyslipidemia profile (*e.g.*, triglyceride reduction).^20^

The EPA/AA ratio is an established indicator of atherosclerotic cardiovascular disease risk,^63^ and recent clinical investigations have explored using this marker to identify patients who would benefit from IPE treatment. (RESPECT‐EPA).^64^ In this trial, a post hoc analysis revealed a significant reduction in risk in patients in whom there was a significant increase in the EPA/AA ratio compared with control patients in whom there was no change in the EPA/AA ratio. Among their numerous functions, EPA and AA serve as precursors to bioactive lipid metabolites, including prostaglandins, thromboxanes, and specialized proresolving mediators.^65^ EPA and AA compete for binding to cyclooxygenase and lipoxygenase enzymes that convert these fatty acids into anti‐ and proinflammatory mediators, respectively. Treatment with IPE not only increases the EPA/AA ratio, but also increases plasma and red blood cell membranes levels of DPA in patients.^66^ This omega 3 fatty acid has its own antiatherosclerotic actions, including conversion to specialized proresolving mediators, preventing cholesterol crystal formation, membrane oxidation, and lipoprotein oxidation in a manner exceeded only by EPA.^27^, ^67^ Together, these data show the interplay of EPA and endothelial function under disease‐like conditions, which may be mediated through direct and indirect actions of EPA, its ratio to AA, and its various lipid products.

Our Gene Set Enrichment Analysis revealed that EPA significantly modulated proteins associated with the pathway denoted “neutrophil degranulation.” This is a proinflammatory process that contributes to complications of atherosclerosis and tissue injury as well as certain inflammatory disorders.^68^, ^69^ Neutrophils play a key role in mediating cellular migration across the endothelium by releasing proteases and pro‐oxidant mediators.^70^ Several studies have linked NO and nitrated lipids with inhibition of neutrophil activities including degranulation.^71^, ^72^, ^73^ EPA decreased the expression of 15 and increased the expression of 17 proteins associated with this pathway relative to IL‐6 alone. This included proteins such as ITGAV. Integrins are a family of cell surface α‐β heterodimer receptors that bind a wide range of ligands, including vitronectin, fibrinogen, platelet endothelial cell adhesion molecular‐1, von Willebrand factor, and matrix metalloproteinases.^74^ αVβ3 (integrin αV:β3) is crucial in IGF‐1 (insulin‐like growth factor‐1) and FGF‐1 (fibroblast growth factor‐1) signaling, both of which are polypeptides involved in tumor progression of certain cancers.^75^, ^76^ Additional studies have shown that αVβ3 is a critical piece in the signaling cascade of the proinflammatory cytokine IL‐1β, and both αVβ3 and αVβ6 mediate acute lung injury caused by IL‐1β.^77^, ^78^ The downregulation of integrin αV may be a novel anti‐inflammatory mechanism of EPA with relevance to cardiovascular disease and other pathologies linked to inflammation such as diabetes. Another mechanism of neutrophil activation is the expression and presentation of HMGB1 on activated platelets, which recruits leukocytes and induces, among other actions, the formation of neutrophil extracellular traps in both animal models and patients following acute myocardial infarction.^79^, ^80^ Sufficient platelet‐derived HMGB1 is thus a critical part of thrombus formation.^81^ Platelets and their nucleated megakaryocyte precursors are known to express functional HMOX‐1,^82^, ^83^ and the EPA concentration in both megakaryocytes and platelets is known to increase following its intake.^84^, ^85^ Thus, it is possible that EPA may regulate HMOX‐1 levels and, by extension, HMGB1 in platelets, thereby contributing to the antithrombotic effects observed with IPE treatment in REDUCE‐IT. Such hypotheses will require future testing.

Limitations of the present study include the application of these results to more complicated biological systems in vivo as these analyses used cultured cells using only one inflammatory initiator. Additional analyses using ECs from other vascular beds (eg, pulmonary, glomerular) are warranted, as is testing other initiators of inflammation (eg, TNF‐α, IL‐1β, high glucose) while measuring gene expression and protein phosphorylation patterns. Additional clinical investigations may also be warranted in other inflammatory diseases that induce an increase in cytokine release in the vasculature, including infections such as influenza.^86^, ^87^

## CONCLUSIONS

Challenge with IL‐6 induced significant endothelial inflammation and dysfunction that was reversed with EPA treatment. This was evidenced by increased NO bioavailability and favorable modulation in expression of anti‐inflammatory and cytoprotective proteins including HMOX‐1. This effect was also independent of changes in eNOS levels. Additionally, EPA treatment significantly modulated pathways related to ROS detoxification and neutrophil degranulation. These data identify potential mechanisms of action of EPA and its protective effects in the endothelium under inflammatory conditions relevant to those in atherosclerotic plaque development, thereby expanding our understanding of the demonstrated clinical benefits of EPA therapy in high‐risk patients.

## Sources of Funding

Dr. Libby receives funding support from the National Heart, Lung, and Blood Institute (1R01HL134892, 1R01HL163099‐01, R01AG063839, R01HL151627, R01HL157073, R01HL166538), the RRM Charitable Fund, and the Simard Fund.

## Disclosures

Dr. Sherratt is an employee of Elucida Research LLC. Dr. Dawoud has no competing interests. Dr. Mason has received consulting or research grants from Amarin Pharma Inc., HLS Therapeutics, Esperion, Lexicon, and the Cleveland Clinic. Dr. Bhatt discloses the following relationships—Advisory Board: Angiowave, Bayer, Boehringer Ingelheim, CellProthera, Cereno Scientific, Elsevier Practice Update Cardiology, High Enroll, Janssen, Level Ex, McKinsey, Medscape Cardiology, Merck, MyoKardia, NirvaMed, Novo Nordisk, PhaseBio, PLx Pharma, Stasys; Board of Directors: American Heart Association New York City, Angiowave (stock options), Bristol Myers Squibb (stock), DRS.LINQ (stock options), High Enroll (stock); Consultant: Broadview Ventures, Hims, SFJ, Youngene; Data Monitoring Committees: Acesion Pharma, Assistance Publique‐Hôpitaux de Paris, Baim Institute for Clinical Research (formerly Harvard Clinical Research Institute, for the PORTICO trial, funded by St. Jude Medical, now Abbott), Boston Scientific (Chair, PEITHO trial), Cleveland Clinic, Contego Medical (Chair, PERFORMANCE 2), Duke Clinical Research Institute, Mayo Clinic, Mount Sinai School of Medicine (for the ENVISAGE trial, funded by Daiichi Sankyo; for the ABILITY‐DM trial, funded by Concept Medical; for ALLAY‐HF, funded by Alleviant Medical), Novartis, Population Health Research Institute; Rutgers University (for the National Institutes of Health‐funded MINT Trial); Honoraria: American College of Cardiology (Senior Associate Editor, Clinical Trials and News, ACC.org; Chair, ACC Accreditation Oversight Committee), Arnold and Porter law firm (work related to Sanofi/Bristol‐Myers Squibb clopidogrel litigation), Baim Institute for Clinical Research (formerly Harvard Clinical Research Institute; RE‐DUAL PCI clinical trial steering committee funded by Boehringer Ingelheim; AEGIS‐II executive committee funded by CSL Behring), Belvoir Publications (Editor in Chief, *Harvard Heart Letter*), Canadian Medical and Surgical Knowledge Translation Research Group (clinical trial steering committees), CSL Behring (American Heart Association lecture), Cowen and Company, Duke Clinical Research Institute (clinical trial steering committees, including for the PRONOUNCE trial, funded by Ferring Pharmaceuticals), HMP Global (Editor in Chief, *Journal of Invasive Cardiology*), *Journal of the American College of Cardiology* (Guest Editor; Associate Editor), K2P (Co‐Chair, interdisciplinary curriculum), Level Ex, Medtelligence/ReachMD (CME steering committees), MJH Life Sciences, Oakstone CME (Course Director, Comprehensive Review of Interventional Cardiology), Piper Sandler, Population Health Research Institute (for the COMPASS operations committee, publications committee, steering committee, and USA national co‐leader, funded by Bayer), WebMD (continuing medical education steering committees), Wiley (steering committee); Other: Clinical Cardiology (Deputy Editor); Patent: Sotagliflozin (named on a patent for sotagliflozin assigned to Brigham and Women's Hospital who assigned to Lexicon; neither I nor Brigham and Women's Hospital receive any income from this patent); Research Funding: Abbott, Acesion Pharma, Afimmune, Aker Biomarine, Alnylam, Amarin, Amgen, AstraZeneca, Bayer, Beren, Boehringer Ingelheim, Boston Scientific, Bristol‐Myers Squibb, Cardax, CellProthera, Cereno Scientific, Chiesi, CinCor, Cleerly, CSL Behring, Eisai, Ethicon, Faraday Pharmaceuticals, Ferring Pharmaceuticals, Forest Laboratories, Fractyl, Garmin, HLS Therapeutics, Idorsia, Ironwood, Ischemix, Janssen, Javelin, Lexicon, Lilly, Medtronic, Merck, Moderna, MyoKardia, NirvaMed, Novartis, Novo Nordisk, Otsuka, Owkin, Pfizer, PhaseBio, PLx Pharma, Recardio, Regeneron, Reid Hoffman Foundation, Roche, Sanofi, Stasys, Synaptic, The Medicines Company, Youngene, 89Bio; Royalties: Elsevier (Editor, *Braunwald's Heart Disease*); Site Co‐Investigator: Abbott, Biotronik, Boston Scientific, CSI, Endotronix, St. Jude Medical (now Abbott), Philips, SpectraWAVE, Svelte, Vascular Solutions; Trustee: American College of Cardiology; Unfunded Research: FlowCo. Dr. Libby is an unpaid consultant to, or involved in clinical trials for Amgen, Baim Institute, Beren Therapeutics, Esperion Therapeutics, Genentech, Kancera, Kowa Pharmaceuticals, Novo Nordisk, Novartis, and Sanofi‐Regeneron. Dr. Libby is a member of the scientific advisory board for Amgen, Caristo Diagnostics, CSL Behring, DalCor Pharmaceuticals, Dewpoint Therapeutics, Eulicid Bioimaging, Kancera, Kowa Pharmaceuticals, Olatec Therapeutics, MedImmune, Novartis, PlaqueTec, Polygon Therapeutics, TenSixteen Bio, Soley Therapeutics, and XBiotech, Inc. Dr. Libby's laboratory has received research funding in the last 2 years from Novartis, Novo Nordisk and Genentech. Dr. Libby is on the Board of Directors of XBiotech, Inc. Dr. Libby has a financial interest in Xbiotech, a company developing therapeutic human antibodies, in TenSixteen Bio, a company targeting somatic mosaicism and clonal hematopoiesis of indeterminate potential (CHIP) to discover and develop novel therapeutics to treat age‐related diseases, and in Soley Therapeutics, a biotechnology company that is combining artificial intelligence with molecular and cellular response detection for discovering and developing new drugs, currently focusing on cancer therapeutics. Dr. Libby's interests were reviewed and managed by Brigham and Women's Hospital and Mass General Brigham in accordance with their conflict‐of‐interest policies.

## Supporting information

Tables S1–S3
Figures S1–S4
