# Supplementary material for: Eicosapentaenoic Acid Improves Endothelial Nitric Oxide Bioavailability Via Changes in Protein Expression During Inflammation
Source: J Am Heart Assoc. 2024 Jul 3;13(14):e034076. doi: 10.1161/JAHA.123.034076 (PMC11292741; doi:10.1161/JAHA.123.034076)
Supplement: Supplementary file 1 — Tables S1–S3 Figures S1–S4 [file JAH3-13-e034076-s001.pdf]

SUPPLEMENTAL MATERIAL

Table S1

Protein	EPA 10 µM (IL-6 vs EPA + IL-6)		EPA 40 µM (IL-6 vs EPA + IL-6)	
	Log2 Fold Change	*p*-value	Log2 Fold Change	*p*-value
**DDAH1**	-0.131	0.046	-0.0937	0.035
**HMGB1**	-0.126	0.025	-0.17	0.0002
**HMOX1**	-0.269	0.002	-1.04	2.12×10^{-9}
**ITGAV**	0.451	0.0006	0.0858	0.010
**ITGB1**	0.346	9.57×10^{-5}	0.169	0.0004
**NQO1**	-0.452	1.66×10^{-6}	-0.377	2.29×10^{-6}
**PARK7**	-0.255	0.001	-0.12	0.003
**PRDX2**	-0.239	0.002	-0.071	0.044
**SNCA**	-0.311	0.009	-0.204	1.99×10^{-5}
**TXN**	-0.539	0.006	-0.238	1.35×10^{-5}

Summary of Log2 Fold Change and P-Values of Anti-Inflammatory and Antioxidant Response Proteins Modulated by EPA relative to IL-6 at 10 and 40 µM. the sign of the fold change value indicates direction of change. Key: DDAH1, dimethylarginine dimethylaminohydrolase-1; HMGB1, high mobility group box protein 1; HMOX1, heme oxygenase-1; ITGAV, integrin αV; ITGB1, integrin B1; NQO1, NAD(P)H quinone oxidoreductase-1; PARK1, Parkinson disease protein 7; PRDX2, peroxiredoxin-2; SNCA; α-synuclein; TXN, thioredoxin.

Table S2 - Full GSEA Summary

IL-6 vs EPA + IL-6

	PATHWAY	COMPARISON	LOG_ODDS	OVERLAP	NOMINAL P.value	ADJUSTED.P.value	Genes
44540	neutrophil degranulation (GO:0043312)	IL6_alone_vs_EPA_IL6	0.122677051	'32/487	8.42E-11	4.01E-07	CSTB;PSMD11;ROCK1;COPB1;MVP;GDI2;UBR4;HMGB1;SRP14;CNN2;NCSTN;ITGAV;LTA4H;CTSD;HMOX2;HSPA8;GSN;DGAT1;PGAM1;EEF2;IGF2R;APRT;SERPINB6;RAB10;PKM;FABP5;PECAM1;HPSE;ACTR10;PPIA;KPNB1;FTL
44542	maturation of LSU-rRNA (GO:0000470)	IL6_alone_vs_EPA_IL6	-0.154103469	'17/203	8.24E-08	8.98E-05	RPL5:UTP4:RPLP1:RPLP0:NOP2:RPL13A:NOC2L:RPL6:NOL6:RSL1D1:RPS17:XRN2:MRTO4:SBDS:RPS11:EXOSC2:RPS23
44544	mRNA trans splicing, via spliceosome (GO:0000365)	IL6_alone_vs_EPA_IL6	-0.130894067	'18/233	1.17E-07	8.98E-05	HSPA8:BUD31:CDC5L:HNRNPR:CWC27:PNN:LSM8:PUF60:SNRPD1:PSPC1:U2AF2:HNRNPF:PRPF3:SNRPA1:SRSF5:SNRNP200:HNRNPA1:PPIL3
44545	spliceosomal conformational changes to generate catalytic conformation (GO:0000393)	IL6_alone_vs_EPA_IL6	-0.130894067	'18/234	1.25E-07	8.98E-05	HSPA8:BUD31:CDC5L:HNRNPR:CWC27:PNN:LSM8:PUF60:SNRPD1:PSPC1:U2AF2:HNRNPF:PRPF3:SNRPA1:SRSF5:SNRNP200:HNRNPA1:PPIL3
44546	spliceosomal complex disassembly (GO:0000390)	IL6_alone_vs_EPA_IL6	-0.130894067	'18/234	1.25E-07	8.98E-05	HSPA8:BUD31:CDC5L:HNRNPR:CWC27:PNN:LSM8:PUF60:SNRPD1:PSPC1:U2AF2:HNRNPF:PRPF3:SNRPA1:SRSF5:SNRNP200:HNRNPA1:PPIL3
44547	SRP-dependent cotranslational protein targeting to membrane (GO:0006614)	IL6_alone_vs_EPA_IL6	-0.375720912	'12/100	1.41E-07	8.98E-05	RPL5;RPS17;SPCS2;ARL6IP1;RPLP1;RPLP0;RPL13A;SRP68;SRP14;RPS11;RPL6;RPS23
44549	mRNA cis splicing, via spliceosome (GO:0045292)	IL6_alone_vs_EPA_IL6	-0.130894067	'18/236	1.42E-07	8.98E-05	HSPA8:BUD31:CDC5L:HNRNPR:CWC27:PNN:LSM8:PUF60:SNRPD1:PSPC1:U2AF2:HNRNPF:PRPF3:SNRPA1:SRSF5:SNRNP200:HNRNPA1:PPIL3
44551	alternative mRNA splicing, via spliceosome (GO:0000380)	IL6_alone_vs_EPA_IL6	-0.16183258	'18/237	1.51E-07	8.98E-05	HSPA8:BUD31:CDC5L:HNRNPR:CWC27:PNN:LSM8:PUF60:SNRPD1:PSPC1:U2AF2:HNRNPF:PRPF3:SNRPA1:SRSF5:SNRNP200:HNRNPA1:PPIL3
44553	spliceosomal complex assembly (GO:0000245)	IL6_alone_vs_EPA_IL6	-0.146436649	'18/241	1.94E-07	0.000102615	HSPA8:BUD31:CDC5L:HNRNPR:CWC27:PNN:LSM8:PUF60:SNRPD1:PSPC1:U2AF2:HNRNPF:PRPF3:SNRPA1:SRSF5:SNRNP200:HNRNPA1:PPIL3
44555	spliceosomal snRNP assembly (GO:0000387)	IL6_alone_vs_EPA_IL6	-0.146436649	'18/246	2.63E-07	0.000125398	HSPA8:BUD31:CDC5L:HNRNPR:CWC27:PNN:LSM8:PUF60:SNRPD1:PSPC1:U2AF2:HNRNPF:PRPF3:SNRPA1:SRSF5:SNRNP200:HNRNPA1:PPIL3
44557	mRNA splicing, via spliceosome (GO:0000398)	IL6_alone_vs_EPA_IL6	-0.19219613	'18/252	3.76E-07	0.000162832	HSPA8:BUD31:CDC5L:HNRNPR:CWC27:PNN:LSM8:PUF60:SNRPD1:PSPC1:U2AF2:HNRNPF:PRPF3:SNRPA1:SRSF5:SNRNP200:HNRNPA1:PPIL3
44559	rRNA modification (GO:0000154)	IL6_alone_vs_EPA_IL6	-0.243019473	'15/187	8.32E-07	0.000329923	RPL5;UTP4;RPLP1;RPLP0;RPL13A;RPL6;LRPPRC;NOL6;RPS17;MRTO4;XRN2;SBDS;RPS11;EXOSC2;RPS23
44561	gluconeogenesis (GO:0006094)	IL6_alone_vs_EPA_IL6	1.515912892	'9/64	1.35E-06	0.000496101	SLC25A1;TPI1;GNPDA1;MDH2;PGAM1;GOT2;AKR1A1;PGK1;GAPDH
44563	negative regulation of anoikis (GO:2000811)	IL6_alone_vs_EPA_IL6	0.56196686	'20/336	1.53E-06	0.000518701	HSPA9;ITGB1;ARF4;ANXA1;ARL6IP1;PRKDC;ANXA4;GLO1;ANXA5;FHL2;PARK7;AATF;HSP90B1;DNAJA1;PRDX2;PTRH2;NUP62;PPIF;ITGA5;SNCA
44565	SRP-dependent cotranslational protein targeting to membrane, signal sequence processing (GO:0006618)	IL6_alone_vs_EPA_IL6	-0.52511694	'11/108	2.45E-06	0.000524167	RPL5;RPS17;SPCS2;RPLP1;RPLP0;RPL13A;SRP68;SRP14;RPS11;RPL6;RPS23
44566	DNA-templated viral transcription (GO:0039695)	IL6_alone_vs_EPA_IL6	-0.544380287	'11/108	2.45E-06	0.000524167	RANBP2;RPL5;RPS17;SEH1L;RPLP1;RPLP0;NUP62;RPL13A;RPS11;RPL6;RPS23
44567	middle viral transcription (GO:0019084)	IL6_alone_vs_EPA_IL6	-0.544380287	'11/108	2.45E-06	0.000524167	RANBP2;RPL5;RPS17;SEH1L;RPLP1;RPLP0;NUP62;RPL13A;RPS11;RPL6;RPS23
44568	RNA-templated viral transcription (GO:0039696)	IL6_alone_vs_EPA_IL6	-0.544380287	'11/108	2.45E-06	0.000524167	RANBP2;RPL5;RPS17;SEH1L;RPLP1;RPLP0;NUP62;RPL13A;RPS11;RPL6;RPS23
44569	negative regulation of nitrosative stress-induced intrinsic apoptotic signaling pathway (GO:1905259)	IL6_alone_vs_EPA_IL6	1.850983713	'5/14	2.50E-06	0.000524167	DNAJA1;NDUFS3;PPIF;PARK7;NOC2L
44570	SRP-dependent cotranslational protein targeting to membrane, docking (GO:0006615)	IL6_alone_vs_EPA_IL6	-0.587489782	'10/88	2.60E-06	0.000524167	RPL5;RPS17;RPLP1;RPLP0;RPL13A;SRP68;SRP14;RPS11;RPL6;RPS23
44571	early viral transcription (GO:0019085)	IL6_alone_vs_EPA_IL6	-0.544380287	'11/110	2.94E-06	0.000524167	RANBP2;RPL5;RPS17;SEH1L;RPLP1;RPLP0;NUP62;RPL13A;RPS11;RPL6;RPS23
44572	chloroplast rRNA processing (GO:1901259)	IL6_alone_vs_EPA_IL6	-0.329582474	'14/181	2.97E-06	0.000524167	RPL5;UTP4;RPLP1;RPLP0;RPL13A;RPL6;NOL6;RPS17;MRTO4;XRN2;SBDS;RPS11;EXOSC2;RPS23
44573	rRNA 5'-end processing (GO:0000967)	IL6_alone_vs_EPA_IL6	-0.329582474	'14/181	2.97E-06	0.000524167	RPL5;UTP4;RPLP1;RPLP0;RPL13A;RPL6;NOL6;RPS17;MRTO4;XRN2;SBDS;RPS11;EXOSC2;RPS23
44574	rRNA 3'-end processing (GO:0031125)	IL6_alone_vs_EPA_IL6	-0.329582474	'14/181	2.97E-06	0.000524167	RPL5;UTP4;RPLP1;RPLP0;RPL13A;RPL6;NOL6;RPS17;MRTO4;XRN2;SBDS;RPS11;EXOSC2;RPS23
44576	maturation of 4.5S rRNA (GO:0000476)	IL6_alone_vs_EPA_IL6	-0.329582474	'14/181	2.97E-06	0.000524167	RPL5;UTP4;RPLP1;RPLP0;RPL13A;RPL6;NOL6;RPS17;MRTO4;XRN2;SBDS;RPS11;EXOSC2;RPS23
44577	snoRNA release from pre-rRNA (GO:1990417)	IL6_alone_vs_EPA_IL6	-0.329582474	'14/181	2.97E-06	0.000524167	RPL5;UTP4;RPLP1;RPLP0;RPL13A;RPL6;NOL6;RPS17;MRTO4;XRN2;SBDS;RPS11;EXOSC2;RPS23
44578	maturation of 2S rRNA (GO:0000475)	IL6_alone_vs_EPA_IL6	-0.329582474	'14/181	2.97E-06	0.000524167	RPL5;UTP4;RPLP1;RPLP0;RPL13A;RPL6;NOL6;RPS17;MRTO4;XRN2;SBDS;RPS11;EXOSC2;RPS23
44579	maturation of 5S rRNA (GO:0000481)	IL6_alone_vs_EPA_IL6	-0.346960326	'14/182	3.17E-06	0.00053923	RPL5;UTP4;RPLP1;RPLP0;RPL13A;RPL6;NOL6;RPS17;MRTO4;XRN2;SBDS;RPS11;EXOSC2;RPS23
44580	late viral transcription (GO:0019086)	IL6_alone_vs_EPA_IL6	-0.544380287	'11/112	3.51E-06	0.000572029	RANBP2;RPL5;RPS17;SEH1L;RPLP1;RPLP0;NUP62;RPL13A;RPS11;RPL6;RPS23
44581	cleavage involved in rRNA processing (GO:0000469)	IL6_alone_vs_EPA_IL6	-0.364152416	'14/184	3.61E-06	0.000572029	RPL5;UTP4;RPLP1;RPLP0;RPL13A;RPL6;NOL6;RPS17;MRTO4;XRN2;SBDS;RPS11;EXOSC2;RPS23
44582	SRP-dependent cotranslational protein targeting to membrane, translocation (GO:0006616)	IL6_alone_vs_EPA_IL6	-0.587489782	'10/92	3.90E-06	0.000599313	RPL5;RPS17;RPLP1;RPLP0;RPL13A;SRP68;SRP14;RPS11;RPL6;RPS23
44584	maturation of SSU-rRNA (GO:0030490)	IL6_alone_vs_EPA_IL6	-0.329582474	'14/186	4.09E-06	0.000608449	RPL5;UTP4;RPLP1;RPLP0;RPL13A;RPL6;NOL6;RPS17;MRTO4;XRN2;SBDS;RPS11;EXOSC2;RPS23
44586	maturation of 5.8S rRNA (GO:0000460)	IL6_alone_vs_EPA_IL6	-0.364152416	'14/187	4.35E-06	0.000628049	RPL5;UTP4;RPLP1;RPLP0;RPL13A;RPL6;NOL6;RPS17;MRTO4;XRN2;SBDS;RPS11;EXOSC2;RPS23
44588	rRNA processing (GO:0006364)	IL6_alone_vs_EPA_IL6	-0.329582474	'14/188	4.63E-06	0.000648598	RPL5;UTP4;RPLP1;RPLP0;RPL13A;RPL6;NOL6;RPS17;MRTO4;XRN2;SBDS;RPS11;EXOSC2;RPS23
44590	viral RNA editing (GO:0075527)	IL6_alone_vs_EPA_IL6	-0.600809257	'11/119	6.34E-06	0.000837779	RANBP2;RPL5;RPS17;SEH1L;RPLP1;RPLP0;NUP62;RPL13A;RPS11;RPL6;RPS23
44591	viral transcription (GO:0019083)	IL6_alone_vs_EPA_IL6	-0.600809257	'11/119	6.34E-06	0.000837779	RANBP2;RPL5;RPS17;SEH1L;RPLP1;RPLP0;NUP62;RPL13A;RPS11;RPL6;RPS23
44593	negative regulation of cysteine-type endopeptidase activity involved in apoptotic process (GO:0043154)	IL6_alone_vs_EPA_IL6	0.483492801	'19/346	8.91E-06	0.001146715	HSPA9;ARF4;ANXA1;ARL6IP1;CSNK2A1;PRKDC;ANXA4;GLO1;ANXA5;FHL2;PARK7;AATF;HSP90B1;DNAJA1;PRDX2;IFI16;NUP62;PPIF;SNCA
44595	SRP-dependent cotranslational protein targeting to membrane, signal sequence recognition (GO:0006617)	IL6_alone_vs_EPA_IL6	-0.607737759	'10/102	9.92E-06	0.001242047	RPL5;RPS17;RPLP1;RPLP0;RPL13A;SRP68;SRP14;RPS11;RPL6;RPS23
44596	negative regulation of neuron apoptotic process (GO:0043524)	IL6_alone_vs_EPA_IL6	0.456632772	'20/399	1.96E-05	0.002396338	HSPA9;ARF4;ANXA1;ARL6IP1;PRKDC;ANXA4;GLO1;ANXA5;FHL2;PARK7;AATF;SOD2;HSP90B1;SOD1;DNAJA1;PRDX2;NUP62;AKT1S1;PPIF;SNCA
44598	translational elongation (GO:0006414)	IL6_alone_vs_EPA_IL6	-0.645817706	'12/162	2.34E-05	0.00278291	EEF1B2;RPL5;RPS17;MRPS15;RPLP1;RPLP0;RPL13A;RPS11;EEF2;MRPL24;RPL6;RPS23
44600	nuclear-transcribed mRNA catabolic process, 3'-5' exonucleolytic nonsense-mediated decay (GO:0070478)	IL6_alone_vs_EPA_IL6	-0.725211121	'11/143	3.61E-05	0.004188113	RPL5;RPS17;CNOT7;RPLP1;RPLP0;RPL13A;PPP2R2A;RPS11;EXOSC2;RPL6;RPS23
44601	endoplasmic reticulum-Golgi intermediate compartment (ERGIC) derived vesicle fusion with Golgi cis cisterna membra	IL6_alone_vs_EPA_IL6	0.232860027	'11/146	4.37E-05	0.004540598	ARF4;DYNC1I2;SEC23A;SEC24B;TFG;COPB1;RAB1B;TMED2;ARFGAP3;SEC31A;ARCN1
44602	vesicle targeting, rough ER to cis-Golgi (GO:0048207)	IL6_alone_vs_EPA_IL6	0.232860027	'11/146	4.37E-05	0.004540598	ARF4;DYNC1I2;SEC23A;SEC24B;TFG;COPB1;RAB1B;TMED2;ARFGAP3;SEC31A;ARCN1
44604	cytoplasmic translational elongation (GO:0002182)	IL6_alone_vs_EPA_IL6	0.650689572	'5/24	4.62E-05	0.004540598	EEF1B2;RPLP1;EIF4H;EEF2;RPL6
44605	COPII vesicle uncoating (GO:0090112)	IL6_alone_vs_EPA_IL6	0.232860027	'11/147	4.65E-05	0.004540598	ARF4;DYNC1I2;SEC23A;SEC24B;TFG;COPB1;RAB1B;TMED2;ARFGAP3;SEC31A;ARCN1
44606	negative regulation of apoptotic process involved in development (GO:1904746)	IL6_alone_vs_EPA_IL6	0.403793704	'17/325	4.87E-05	0.004540598	HSPA9;ARF4;ANXA1;ARL6IP1;PRKDC;ANXA4;GLO1;ANXA5;FHL2;PARK7;AATF;HSP90B1;DNAJA1;PRDX2;NUP62;PPIF;SNCA
44607	negative regulation of fat cell apoptotic process (GO:1904650)	IL6_alone_vs_EPA_IL6	0.403793704	'17/325	4.87E-05	0.004540598	HSPA9;ARF4;ANXA1;ARL6IP1;PRKDC;ANXA4;GLO1;ANXA5;FHL2;PARK7;AATF;HSP90B1;DNAJA1;PRDX2;NUP62;PPIF;SNCA
44608	negative regulation of epithelial cell apoptotic process (GO:1904036)	IL6_alone_vs_EPA_IL6	0.403793704	'17/325	4.87E-05	0.004540598	HSPA9;ARF4;ANXA1;ARL6IP1;PRKDC;ANXA4;GLO1;ANXA5;FHL2;PARK7;AATF;HSP90B1;DNAJA1;PRDX2;NUP62;PPIF;SNCA
44610	negative regulation of myofibroblast cell apoptotic process (GO:1904521)	IL6_alone_vs_EPA_IL6	0.403793704	'17/325	4.87E-05	0.004540598	HSPA9;ARF4;ANXA1;ARL6IP1;PRKDC;ANXA4;GLO1;ANXA5;FHL2;PARK7;AATF;HSP90B1;DNAJA1;PRDX2;NUP62;PPIF;SNCA
44611	negative regulation of apoptotic process in bone marrow (GO:0071866)	IL6_alone_vs_EPA_IL6	0.403793704	'17/325	4.87E-05	0.004540598	HSPA9;ARF4;ANXA1;ARL6IP1;PRKDC;ANXA4;GLO1;ANXA5;FHL2;PARK7;AATF;HSP90B1;DNAJA1;PRDX2;NUP62;PPIF;SNCA
44612	negative regulation of compound eye retinal cell apoptotic process (GO:1901693)	IL6_alone_vs_EPA_IL6	0.403793704	'17/325	4.87E-05	0.004540598	HSPA9;ARF4;ANXA1;ARL6IP1;PRKDC;ANXA4;GLO1;ANXA5;FHL2;PARK7;AATF;HSP90B1;DNAJA1;PRDX2;NUP62;PPIF;SNCA
44613	negative regulation of fibroblast apoptotic process (GO:2000270)	IL6_alone_vs_EPA_IL6	0.403793704	'17/326	5.06E-05	0.004540598	HSPA9;ARF4;ANXA1;ARL6IP1;PRKDC;ANXA4;GLO1;ANXA5;FHL2;PARK7;AATF;HSP90B1;DNAJA1;PRDX2;NUP62;PPIF;SNCA
44614	negative regulation of muscle cell apoptotic process (GO:0010656)	IL6_alone_vs_EPA_IL6	0.403793704	'17/326	5.06E-05	0.004540598	HSPA9;ARF4;ANXA1;ARL6IP1;PRKDC;ANXA4;GLO1;ANXA5;FHL2;PARK7;AATF;HSP90B1;DNAJA1;PRDX2;NUP62;PPIF;SNCA
44615	negative regulation of myeloid cell apoptotic process (GO:0033033)	IL6_alone_vs_EPA_IL6	0.403793704	'17/327	5.25E-05	0.004544902	HSPA9;ARF4;ANXA1;ARL6IP1;PRKDC;ANXA4;GLO1;ANXA5;FHL2;PARK7;AATF;HSP90B1;DNAJA1;PRDX2;NUP62;PPIF;SNCA
44616	negative regulation of mesenchymal cell apoptotic process (GO:2001054)	IL6_alone_vs_EPA_IL6	0.403793704	'17/327	5.25E-05	0.004544902	HSPA9;ARF4;ANXA1;ARL6IP1;PRKDC;ANXA4;GLO1;ANXA5;FHL2;PARK7;AATF;HSP90B1;DNAJA1;PRDX2;NUP62;PPIF;SNCA
44617	COPII-coated vesicle budding (GO:0090114)	IL6_alone_vs_EPA_IL6	0.232860027	'11/150	5.59E-05	0.004657765	ARF4;DYNC1I2;SEC23A;SEC24B;TFG;COPB1;RAB1B;TMED2;ARFGAP3;SEC31A;ARCN1
44619	nuclear-transcribed mRNA catabolic process, nonsense-mediated decay (GO:0000184)	IL6_alone_vs_EPA_IL6	-0.814928807	'10/125	5.84E-05	0.004657765	RPL5;RPS17;RPLP1;MRTO4;RPLP0;RPL13A;PPP2R2A;RPS11;RPL6;RPS23
44621	negative regulation of execution phase of apoptosis (GO:1900118)	IL6_alone_vs_EPA_IL6	0.403793704	'17/330	5.88E-05	0.004657765	HSPA9;ARF4;ANXA1;ARL6IP1;PRKDC;ANXA4;GLO1;ANXA5;FHL2;PARK7;AATF;HSP90B1;DNAJA1;PRDX2;NUP62;PPIF;SNCA
44623	negative regulation of glial cell apoptotic process (GO:0034351)	IL6_alone_vs_EPA_IL6	0.403793704	'17/330	5.88E-05	0.004657765	HSPA9;ARF4;ANXA1;ARL6IP1;PRKDC;ANXA4;GLO1;ANXA5;FHL2;PARK7;AATF;HSP90B1;DNAJA1;PRDX2;NUP62;PPIF;SNCA
44624	endoplasmic reticulum-derived vesicle fusion with endoplasmic reticulum-Golgi intermediate compartment (ERGIC) m	IL6_alone_vs_EPA_IL6	0.201618069	'11/151	5.94E-05	0.004657765	ARF4;DYNC1I2;SEC23A;SEC24B;TFG;COPB1;RAB1B;TMED2;ARFGAP3;SEC31A;ARCN1
44625	negative regulation of apoptotic process (GO:0043066)	IL6_alone_vs_EPA_IL6	0.321832917	'21/467	5.97E-05	0.004657765	HSPA9;ARF4;ANXA1;ARL6IP1;PRKDC;ANXA4;GLO1;ANXA5;FHL2;PARK7;PHB;AATF;HSP90B1;DNAJA1;PRDX2;RSL1D1;PSMC5;NUP62;AKT1S1;PPIF;SNCA
44627	negative regulation of leukocyte apoptotic process (GO:2000107)	IL6_alone_vs_EPA_IL6	0.403793704	'17/331	6.10E-05	0.004685648	HSPA9;ARF4;ANXA1;ARL6IP1;PRKDC;ANXA4;GLO1;ANXA5;FHL2;PARK7;AATF;HSP90B1;DNAJA1;PRDX2;NUP62;PPIF;SNCA
44628	ER to Golgi vesicle-mediated transport (GO:0006888)	IL6_alone_vs_EPA_IL6	0.232860027	'11/152	6.31E-05	0.004767641	ARF4;DYNC1I2;SEC23A;SEC24B;TFG;COPB1;RAB1B;TMED2;ARFGAP3;SEC31A;ARCN1
44630	negative regulation of apoptotic signaling pathway (GO:2001234)	IL6_alone_vs_EPA_IL6	0.403793704	'17/338	7.89E-05	0.005868281	HSPA9;ARF4;ANXA1;ARL6IP1;PRKDC;ANXA4;GLO1;ANXA5;FHL2;PARK7;AATF;HSP90B1;DNAJA1;PRDX2;NUP62;PPIF;SNCA
44632	canonical glycolysis (GO:0061621)	IL6_alone_vs_EPA_IL6	1.069131663	'5/27	8.41E-05	0.00609727	TPI1;PKM;PGAM1;PGK1;GAPDH
44634	cytoplasmic translation (GO:0002181)	IL6_alone_vs_EPA_IL6	-0.673073258	'11/157	8.45E-05	0.00609727	RPL5;RPS17;MRPS15;RPLP1;RPLP0;EIF4H;RPL13A;RPS11;MRPL24;RPL6;RPS23
44636	regulation of transcription from RNA polymerase II promoter in response to oxidative stress (GO:0043619)	IL6_alone_vs_EPA_IL6	0.690115941	'8/83	8.71E-05	0.006190751	PRDX2;TXNL1;HMOX1;PARK7;TXN;SOD2;SNCA;SOD1
44638	negative regulation of actin filament bundle assembly (GO:0032232)	IL6_alone_vs_EPA_IL6	1.915377061	'3/6	9.54E-05	0.006678619	HSPA8;RDX;PFN1
44640	actin filament capping (GO:0051693)	IL6_alone_vs_EPA_IL6	1.748020783	'4/15	9.88E-05	0.006717781	LIMA1;GSN;TWF1;PFN1
44642	sequestering of actin monomers (GO:0042989)	IL6_alone_vs_EPA_IL6	1.748020783	'4/15	9.88E-05	0.006717781	GSN;TMSB4X;TWF1;PFN1
44644	nuclear-transcribed mRNA catabolic process, 5'-3' exonucleolytic nonsense-mediated decay (GO:0070479)	IL6_alone_vs_EPA_IL6	-0.880418384	'9/111	0.000123084	0.008251804	RPL5;RPS17;RPLP1;RPLP0;RPL13A;PPP2R2A;RPS11;RPL6;RPS23
44645	viral translational termination-reinitiation (GO:0075525)	IL6_alone_vs_EPA_IL6	0.82969609	'6/46	0.0001259	0.008323384	RANBP2;SEH1L;EIF3L;NUP62;EIF3D;EIF3A
44646	negative regulation of oxidative stress-induced intrinsic apoptotic signaling pathway (GO:1902176)	IL6_alone_vs_EPA_IL6	1.486717343	'5/30	0.000142357	0.009282441	NDUFS3;PPIF;PARK7;NOC2L;SOD2
44648	IRES-dependent viral translational initiation (GO:0075522)	IL6_alone_vs_EPA_IL6	0.592670596	'6/48	0.000160278	0.01030976	RANBP2;SEH1L;NUP62;EIF3F;EIF3D;EIF3A
44650	negative regulation of hypoxia-induced intrinsic apoptotic signaling pathway (GO:1903298)	IL6_alone_vs_EPA_IL6	1.160514593	'4/17	0.000167653	0.010640373	DDAH1;NDUFS3;PPIF;NOC2L
44651	heterotypic cell-cell adhesion (GO:0034113)	IL6_alone_vs_EPA_IL6	2.334676588	'4/18	0.00021266	0.013319218	ITGB1;PARVA;ITGAV;ITGA5
44653	canonical Wnt signaling pathway involved in negative regulation of apoptotic process (GO:0044336)	IL6_alone_vs_EPA_IL6	0.380889579	'17/371	0.000240131	0.014844469	HSPA9;ARF4;ANXA1;ARL6IP1;PRKDC;ANXA4;GLO1;ANXA5;FHL2;PARK7;AATF;HSP90B1;DNAJA1;PRDX2;NUP62;PPIF;SNCA
44654	Purkinje myocyte-ventricular cardiac muscle cell adhesion involved in cell communication (GO:0086074)	IL6_alone_vs_EPA_IL6	2.334676588	'4/19	0.000265755	0.01612295	ITGB1;PARVA;ITGAV;ITGA5
44655	tRNA aminoacylation for protein translation (GO:0006418)	IL6_alone_vs_EPA_IL6	-0.759054536	'11/179	0.000268447	0.01612295	RPL5;AIMP2;RPS17;MRPS15;RPLP1;RPLP0;RPL13A;RPS11;MRPL24;RPL6;RPS23
44657	pre-miRNA export from nucleus (GO:0035281)	IL6_alone_vs_EPA_IL6	0.380412125	'7/74	0.000270974	0.01612295	RANBP2;CNOT7;U2AF2;XPO5;NUP62;SRSF5;HNRNPA1
44658	plastid translation (GO:0032544)	IL6_alone_vs_EPA_IL6	-0.797166355	'10/152	0.000294336	0.017085873	RPL5;RPS17;MRPS15;RPLP1;RPLP0;RPL13A;RPS11;MRPL24;RPL6;RPS23
44659	maintenance of translational fidelity (GO:1990145)	IL6_alone_vs_EPA_IL6	-0.797166355	'10/152	0.000294336	0.017085873	RPL5;RPS17;MRPS15;RPLP1;RPLP0;RPL13A;RPS11;MRPL24;RPL6;RPS23
44660	negative regulation of histone ubiquitination (GO:0033183)	IL6_alone_vs_EPA_IL6	0.95280405	'5/35	0.000302372	0.017340869	DNAJA1;UBXN1;U2AF2;UBE2N;PARK7
44661	N-terminal peptidyl-proline dimethylation involved in translation (GO:0071891)	IL6_alone_vs_EPA_IL6	-0.797166355	'10/153	0.000310236	0.01758006	RPL5;RPS17;MRPS15;RPLP1;RPLP0;RPL13A;RPS11;MRPL24;RPL6;RPS23
44662	atrial cardiac muscle cell-AV node cell adhesion involved in cell communication (GO:0086071)	IL6_alone_vs_EPA_IL6	2.334676588	'4/20	0.000327738	0.018139921	ITGB1;PARVA;ITGAV;ITGA5
44663	SA node cell-atrial cardiac muscle cell adhesion involved in cell communication (GO:0086022)	IL6_alone_vs_EPA_IL6	2.334676588	'4/20	0.000327738	0.018139921	ITGB1;PARVA;ITGAV;ITGA5
44664	age-dependent response to oxidative stress involved in chronological cell aging (GO:0001324)	IL6_alone_vs_EPA_IL6	0.552908012	'7/78	0.000375317	0.02000067	PRDX2;TXNL1;PARK7;TXN;SOD2;SNCA;SOD1
44665	age-dependent response to oxidative stress involved in replicative cell aging (GO:0001322)	IL6_alone_vs_EPA_IL6	0.552908012	'7/78	0.000375317	0.02000067	PRDX2;TXNL1;PARK7;TXN;SOD2;SNCA;SOD1
44666	cellular response to oxidative stress (GO:0034599)	IL6_alone_vs_EPA_IL6	0.552908012	'7/78	0.000375317	0.02000067	PRDX2;TXNL1;PARK7;TXN;SOD2;SNCA;SOD1
44668	regulation of actin cytoskeleton organization (GO:0032956)	IL6_alone_vs_EPA_IL6	1.112359714	'6/56	0.000378164	0.02000067	CNN2;CLIC4;ROCK1;STMN1;FSCN1;SH3BGRL3
44670	negative regulation of ubiquitin-specific protease activity (GO:2000157)	IL6_alone_vs_EPA_IL6	2.331260468	'3/9	0.000385679	0.020173995	CSTB;UBXN1;PARK7
44671	AV node cell-bundle of His cell adhesion involved in cell communication (GO:0086072)	IL6_alone_vs_EPA_IL6	2.334676588	'4/21	0.000399426	0.020665972	ITGB1;PARVA;ITGAV;ITGA5
44672	translational termination (GO:0006415)	IL6_alone_vs_EPA_IL6	-0.84987458	'10/159	0.000421457	0.021571334	RPL5;RPS17;MRPS15;RPLP1;RPLP0;RPL13A;RPS11;MRPL24;RPL6;RPS23
44674	endoplasmic reticulum-Golgi intermediate compartment (ERGIC) derived vesicle fusion with endoplasmic reticulum me	IL6_alone_vs_EPA_IL6	0.49315787	'7/80	0.00043836	0.021964143	ARF4;RAB1B;COPB1;LMAN2;TMED2;ARFGAP3;ARCN1
44675	regulation of translation in response to oxidative stress (GO:0043556)	IL6_alone_vs_EPA_IL6	0.552908012	'7/80	0.00043836	0.021964143	PRDX2;TXNL1;PARK7;TXN;SOD2;SNCA;SOD1
44676	negative regulation of hydrogen peroxide-mediated programmed cell death (GO:1901299)	IL6_alone_vs_EPA_IL6	2.011902089	'4/22	0.000481648	0.023881706	PSMC5;NUP62;TXN;PARK7
44677	regulation of actin filament bundle assembly (GO:0032231)	IL6_alone_vs_EPA_IL6	1.486717343	'5/39	0.000507129	0.024501144	ROCK1;RDX;FSCN1;SH3BGRL3;PARK7
44679	vesicle targeting, cis-Golgi to rough ER (GO:0048206)	IL6_alone_vs_EPA_IL6	0.49315787	'7/82	0.000509583	0.024501144	ARF4;RAB1B;COPB1;LMAN2;TMED2;ARFGAP3;ARCN1
44680	cellular response to hydroperoxide (GO:0071447)	IL6_alone_vs_EPA_IL6	0.552908012	'7/82	0.000509583	0.024501144	PRDX2;TXNL1;PARK7;TXN;SOD2;SNCA;SOD1
44681	regulation of translation by machinery localization (GO:0043143)	IL6_alone_vs_EPA_IL6	-0.824686533	'11/194	0.000531974	0.025321981	RPL5;MTPN;RPS17;MRPS15;RPLP1;RPLP0;RPL13A;RPS11;MRPL24;RPL6;RPS23
44682	cellular response to sodium arsenite (GO:1903936)	IL6_alone_vs_EPA_IL6	1.915377061	'3/10	0.000544001	0.025638077	GSTO1;PPIF;HNRNPA1
44683	Golgi vesicle fusion with endoplasmic reticulum-Golgi intermediate compartment (ERGIC) membrane (GO:1990688)	IL6_alone_vs_EPA_IL6	0.43571738	'7/84	0.000589728	0.027520643	ARF4;RAB1B;COPB1;LMAN2;TMED2;ARFGAP3;ARCN1
44684	regulation of macroautophagy (GO:0016241)	IL6_alone_vs_EPA_IL6	0.47256023	'8/110	0.000607959	0.028095981	UCHL1;IFI16;EXOC7;USP10;ATP6V1H;HMGB1;ATP6V0D1;GAPDH
44686	mitochondrial translation (GO:0032543)	IL6_alone_vs_EPA_IL6	-0.832496728	'10/167	0.000619577	0.02835758	RPL5;RPS17;MRPS15;RPLP1;RPLP0;RPL13A;RPS11;MRPL24;RPL6;RPS23
44688	tRNA export from nucleus (GO:0006409)	IL6_alone_vs_EPA_IL6	0.275602159	'7/85	0.000633389	0.028713637	RANBP2;SEH1L;U2AF2;NUP62;SRSF5;HNRNPA1;NOL6
44690	oxidative stress-induced premature senescence (GO:0090403)	IL6_alone_vs_EPA_IL6	0.552908012	'7/86	0.000679576	0.0302316	PRDX2;TXNL1;PARK7;TXN;SOD2;SNCA;SOD1
44691	retrograde vesicle-mediated transport, Golgi to ER (GO:0006890)	IL6_alone_vs_EPA_IL6	0.49315787	'7/86	0.000679576	0.0302316	ARF4;RAB1B;COPB1;LMAN2;TMED2;ARFGAP3;ARCN1
44693	regulation of autophagosome assembly (GO:2000785)	IL6_alone_vs_EPA_IL6	0.327086724	'7/87	0.000728391	0.031220497	UCHL1;EXOC7;RAB1B;RDX;ATP6V1H;ATP6V0D1;GAPDH
44695	regulation of mRNA stability involved in cellular response to UV (GO:1902629)	IL6_alone_vs_EPA_IL6	-0.702539761	'9/142	0.000767267	0.031220497	HSPA8;CARHSP1;PSMC5;PSMD11;PCNA;RUVBL2;IGF2BP2;NOC2L;EXOSC2
44696	alternative respiration (GO:0010230)	IL6_alone_vs_EPA_IL6	1.331287989	'4/25	0.000799933	0.031220497	MDH2;UQCRC1;UQCRC2;COX20
44697	bundle of His cell-Purkinje myocyte adhesion involved in cell communication (GO:0086073)	IL6_alone_vs_EPA_IL6	2.011902089	'4/25	0.000799933	0.031220497	ITGB1;ITGAV;PARVA;ITGA5
44699	aerobic respiration, using hydrogen as electron donor (GO:0019412)	IL6_alone_vs_EPA_IL6	1.331287989	'4/25	0.000799933	0.031220497	MDH2;UQCRC1;UQCRC2;COX20
44700	aerobic respiration, using sulfur or sulfate as electron donor (GO:0019414)	IL6_alone_vs_EPA_IL6	1.331287989	'4/25	0.000799933	0.031220497	MDH2;UQCRC1;UQCRC2;COX20
44701	aerobic respiration, using arsenite as electron donor (GO:0043554)	IL6_alone_vs_EPA_IL6	1.331287989	'4/25	0.000799933	0.031220497	MDH2;UQCRC1;UQCRC2;COX20
44703	aerobic respiration, using ferrous ions as electron donor (GO:0019411)	IL6_alone_vs_EPA_IL6	1.331287989	'4/25	0.000799933	0.031220497	MDH2;UQCRC1;UQCRC2;COX20
44704	aerobic respiration, using carbon monoxide as electron donor (GO:0019410)	IL6_alone_vs_EPA_IL6	1.331287989	'4/25	0.000799933	0.031220497	MDH2;UQCRC1;UQCRC2;COX20
44705	aerobic respiration, using nitrite as electron donor (GO:0019332)	IL6_alone_vs_EPA_IL6	1.331287989	'4/25	0.000799933	0.031220497	MDH2;UQCRC1;UQCRC2;COX20
44706	extracellular matrix organization in marginal zone involved in cerebral cortex radial glia guided migration (GO:0021820	IL6_alone_vs_EPA_IL6	0.986373499	'9/143	0.000806748	0.031220497	ITGB1;VWF;LAMA4;BSG;BGN;PECAM1;ITGAV;LAMB1;ITGA5
44707	cellulose microfibril organization (GO:0010215)	IL6_alone_vs_EPA_IL6	0.986373499	'9/143	0.000806748	0.031220497	ITGB1;VWF;LAMA4;BSG;BGN;PECAM1;ITGAV;LAMB1;ITGA5
44708	extracellular matrix organization involved in endocardium development (GO:0061148)	IL6_alone_vs_EPA_IL6	0.986373499	'9/143	0.000806748	0.031220497	ITGB1;VWF;LAMA4;BSG;BGN;PECAM1;ITGAV;LAMB1;ITGA5
44709	extracellular matrix organization (GO:0030198)	IL6_alone_vs_EPA_IL6	0.986373499	'9/143	0.000806748	0.031220497	ITGB1;VWF;LAMA4;BSG;BGN;PECAM1;ITGAV;LAMB1;ITGA5
44711	biofilm matrix organization (GO:0098784)	IL6_alone_vs_EPA_IL6	0.986373499	'9/143	0.000806748	0.031220497	ITGB1;VWF;LAMA4;BSG;BGN;PECAM1;ITGAV;LAMB1;ITGA5
44712	activation of cysteine-type endopeptidase activity involved in apoptotic process (GO:0006919)	IL6_alone_vs_EPA_IL6	0.894217654	'7/89	0.000834332	0.032027575	FIS1;DAP;DIABLO;RACK1;HMGB1;ANP32B;SNCA
44714	fibronectin fibril organization (GO:1905590)	IL6_alone_vs_EPA_IL6	0.986373499	'9/144	0.000847868	0.032030558	ITGB1;VWF;LAMA4;BSG;BGN;PECAM1;ITGAV;LAMB1;ITGA5
44715	basement membrane organization (GO:0071711)	IL6_alone_vs_EPA_IL6	0.986373499	'9/144	0.000847868	0.032030558	ITGB1;VWF;LAMA4;BSG;BGN;PECAM1;ITGAV;LAMB1;ITGA5
44717	tRNA threonylcarbamoyladenosine metabolic process (GO:0070525)	IL6_alone_vs_EPA_IL6	0.121150179	'5/44	0.000892133	0.033176212	TP53RK;HNRNPF;XRN2;HNRNPR;HNRNPA1
44718	aerobic respiration (GO:0009060)	IL6_alone_vs_EPA_IL6	1.195512439	'5/44	0.000892133	0.033176212	MDH2;NDUFS3;UQCRC1;UQCRC2;COX20
44720	negative regulation of nitric-oxide synthase activity (GO:0051001)	IL6_alone_vs_EPA_IL6	1.007662606	'4/26	0.00093274	0.034243588	LYPLA1;SPR;DDAH1;SNCA
44721	gene expression involved in extracellular matrix organization (GO:1901148)	IL6_alone_vs_EPA_IL6	0.986373499	'9/146	0.000935224	0.034243588	ITGB1;VWF;LAMA4;BSG;BGN;PECAM1;ITGAV;LAMB1;ITGA5
44722	RNA modification (GO:0009451)	IL6_alone_vs_EPA_IL6	0.121150179	'5/45	0.000989768	0.035691634	HNRNPF;XRN2;HNRNPR;HNRNPA1;LRPPRC
44724	glycolytic process (GO:0006096)	IL6_alone_vs_EPA_IL6	0.479059257	'5/45	0.000989768	0.035691634	TPI1;GLO1;PGAM1;BSG;PGK1
44726	positive regulation of transcription factor import into nucleus in response to oxidative stress (GO:0036283)	IL6_alone_vs_EPA_IL6	0.327086724	'7/92	0.001015656	0.036349785	PRDX2;TXNL1;PARK7;TXN;SOD2;SNCA;SOD1
44727	translation (GO:0006412)	IL6_alone_vs_EPA_IL6	-0.570584365	'15/348	0.001026861	0.036476569	RPL5;MRPS15;GSN;RPLP1;RPLP0;RPL13A;LAMB1;MRPL24;RPL6;HSP90B1;RPS17;LTA4H;RPS11;RPS23;SNCA
44729	extracellular matrix assembly (GO:0085029)	IL6_alone_vs_EPA_IL6	0.986373499	'9/149	0.001079833	0.037811495	ITGB1;VWF;LAMA4;BSG;BGN;PECAM1;ITGAV;LAMB1;ITGA5
44730	negative regulation of endoplasmic reticulum stress-induced intrinsic apoptotic signaling pathway (GO:1902236)	IL6_alone_vs_EPA_IL6	1.331287989	'4/27	0.001080328	0.037811495	NDUFS3;PPIF;PARK7;NOC2L
44732	protein homooligomerization (GO:0051260)	IL6_alone_vs_EPA_IL6	0.646811157	'9/150	0.001131871	0.039326338	FIS1;STOML2;SNX2;EHD4;VWF;ANXA6;HMOX1;AKR1B1;MICU1
44734	negative regulation of protein ubiquitination involved in ubiquitin-dependent protein catabolic process (GO:2000059)	IL6_alone_vs_EPA_IL6	0.562374326	'5/47	0.001208553	0.041686327	DNAJA1;RPL5;UBXN1;U2AF2;PARK7
44736	stress-induced mitochondrial fusion (GO:1990046)	IL6_alone_vs_EPA_IL6	1.592602563	'3/13	0.001248034	0.042738441	FIS1;STOML2;CHCHD3
44737	mRNA destabilization (GO:0061157)	IL6_alone_vs_EPA_IL6	-0.803814564	'9/153	0.00130027	0.04420919	HSPA8;CARHSP1;PSMC5;PSMD11;RACK1;RPL13A;IGF2BP2;GAPDH;EXOSC2
44739	collagen fibril organization (GO:0030199)	IL6_alone_vs_EPA_IL6	0.864366868	'9/154	0.00136069	0.045935345	ITGB1;VWF;LAMA4;BSG;BGN;PECAM1;ITGAV;LAMB1;ITGA5
44741	positive regulation of transcription regulatory region DNA binding (GO:2000679)	IL6_alone_vs_EPA_IL6	1.331287989	'4/29	0.001423277	0.047709846	HMGB2;TXN;PARK7;HMGB1
44743	positive regulation of wound healing, spreading of epidermal cells (GO:1903691)	IL6_alone_vs_EPA_IL6	0.92411701	'9/156	0.001488313	0.04954105	SUN2;ANXA1;GSN;PODXL;RDX;RACK1;CDH13;LAMB1;SOD2
50994	interleukin-12-mediated signaling pathway (GO:0035722)	IL6_alone_vs_EPA_IL6	4.95588763	'12/46	1.19E-11	1.29E-08	CNN2;HSPA9;RALA;GSTO1;HNRNPF;RPLP0;TALDO1;SNRPA1;SOD2;PPIA;LMNB1;SOD1
50996	cellular response to interleukin-12 (GO:0071349)	IL6_alone_vs_EPA_IL6	4.95588763	'12/46	1.19E-11	1.29E-08	CNN2;HSPA9;RALA;GSTO1;HNRNPF;RPLP0;TALDO1;SNRPA1;SOD2;PPIA;LMNB1;SOD1
51000	neutrophil activation involved in immune response (GO:0002283)	IL6_alone_vs_EPA_IL6	0.144153099	'32/483	6.82E-11	3.66E-08	CSTB;PSMD11;ROCK1;COPB1;MVP;GDI2;UBR4;HMGB1;SRP14;CNN2;NCSTN;ITGAV;LTA4H;CTSD;HMOX2;HSPA8;GSN;DGAT1;PGAM1;EEF2;IGF2R;APRT;SERPINB6;RAB10;PKM;FABP5;PECAM1;HPSE;ACTR10;PPIA;KPNB1;FTL
51001	neutrophil mediated immunity (GO:0002446)	IL6_alone_vs_EPA_IL6	6.461122938	'32/487	8.42E-11	3.66E-08	CSTB;PSMD11;ROCK1;COPB1;MVP;GDI2;UBR4;HMGB1;SRP14;CNN2;NCSTN;ITGAV;LTA4H;CTSD;HMOX2;HSPA8;GSN;DGAT1;PGAM1;EEF2;IGF2R;APRT;SERPINB6;RAB10;PKM;FABP5;PECAM1;HPSE;ACTR10;PPIA;KPNB1;FTL
51003	viral process (GO:0016032)	IL6_alone_vs_EPA_IL6	3.018491953	'20/220	1.41E-09	5.11E-07	RANBP2;RPL5;SEH1L;CTBP2;RPLP1;RAB1B;RPLP0;RPL13A;RPL6;RPS17;EIF3L;NUP62;ELMO1;ATP6V1H;EIF3F;RPS11;PPIA;EIF3D;EIF3A;RPS23
51005	RNA splicing, via transesterification reactions with bulged adenosine as nucleophile (GO:0000377)	IL6_alone_vs_EPA_IL6	3.238617041	'18/236	1.42E-07	4.40E-05	HSPA8:BUD31:CDC5L:HNRNPR:CWC27:PNN:LSM8:PUF60:SNRPD1:PSPC1:U2AF2:HNRNPF:PRPF3:SNRPA1:SRSF5:SNRNP200:HNRNPA1:PPIL3
51009	cotranslational protein targeting to membrane (GO:0006613)	IL6_alone_vs_EPA_IL6	3.014404611	'11/93	5.44E-07	0.000131351	RPL5;RPS17;ARL6IP1;RPLP1;RPLP0;RPL13A;SRP68;SRP14;RPS11;RPL6;RPS23
51013	hexose biosynthetic process (GO:0019319)	IL6_alone_vs_EPA_IL6	1.536010343	'8/44	6.98E-07	0.000137981	SLC25A1:TPI1:MDH2:PGAM1:GOT2:PGK1:AKR1B1:GAPDH
51014	protein targeting to ER (GO:0045047)	IL6_alone_vs_EPA_IL6	2.650138241	'11/97	8.35E-07	0.000151254	RPL5;RPS17;SPCS2;RPLP1;RPLP0;RPL13A;SRP68;SRP14;RPS11;RPL6;RPS23
51016	cellular protein metabolic process (GO:0044267)	IL6_alone_vs_EPA_IL6	2.596329799	'25/484	1.04E-06	0.000174332	RPL5;MRPS15;STOML2;PRKDC;RPLP1;RPLP0;TXN;RPL6;HSP90B1;PCMT1;RPS17;NCSTN;LTA4H;RPS11;SNCA;YES1;GSN;RPL13A;LAMB1;MRPL24;AFG3L2;RSL1D1;SPCS2;UBE2N;RPS23
51018	mRNA processing (GO:0006397)	IL6_alone_vs_EPA_IL6	1.176426567	'18/283	2.02E-06	0.000313132	HSPA8:BUD31:CDC5L:HNRNPR:CWC27:PNN:LSM8:PUF60:SNRPD1:PSPC1:U2AF2:HNRNPF:PRPF3:SNRPA1:SRSF5:SNRNP200:HNRNPA1:PPIL3
51026	viral gene expression (GO:0019080)	IL6_alone_vs_EPA_IL6	2.116224948	'11/110	2.94E-06	0.000354754	RANBP2;RPL5;RPS17;SEH1L;RPLP1;RPLP0;NUP62;RPL13A;RPS11;RPL6;RPS23
51030	regulation of translation (GO:0006417)	IL6_alone_vs_EPA_IL6	2.697552119	'15/213	4.22E-06	0.000459069	RANBP2;RPL5;MTPN;SEH1L;RPL13A;EEF2;EIF5;CNOT7;NUP62;RACK1;EIF4H;IGF2BP2;VIM;GAPDH;EIF3D
51032	cytokine-mediated signaling pathway (GO:0019221)	IL6_alone_vs_EPA_IL6	5.248290197	'28/633	4.80E-06	0.000496532	ITGB1;RALA;PSMD11;RPLP0;HSP90B1;LMNB1;CNN2;MT2A;TMSB4X;HMOX1;TRIM22;STAT5A;HSPA9;HSPA8;ANXA1;GSTO1;BGN;TALDO1;SOD2;SOD1;PSMC5;HNRNPF;FSCN1;UBE2N;SNRPA1;VIM;PPIA;LIMS1
51036	ribosome biogenesis (GO:0042254)	IL6_alone_vs_EPA_IL6	4.705183201	'15/226	8.66E-06	0.000818873	RPL5;UTP4;RPLP1;RPLP0;RPL13A;NOC2L;RPL6;NOL6;RPS17;XRN2;MRTO4;SBDS;RPS11;EXOSC2;RPS23
51038	rRNA metabolic process (GO:0016072)	IL6_alone_vs_EPA_IL6	-0.346960326	'14/200	9.45E-06	0.000855901	RPL5;UTP4;RPLP1;RPLP0;RPL13A;RPL6;NOL6;RPS17;XRN2;MRTO4;SBDS;RPS11;EXOSC2;RPS23
51041	glucose metabolic process (GO:0006006)	IL6_alone_vs_EPA_IL6	3.351851731	'8/64	1.30E-05	0.001083193	SLC25A1:TPI1:MDH2:PGAM1:GOT2:AKR1A1:PGK1:GAPDH
51043	nucleotide-sugar biosynthetic process (GO:0009226)	IL6_alone_vs_EPA_IL6	2.074223533	'5/19	1.36E-05	0.001091498	UGDH;NAGK;GNPDA1;GFPT1;NANS
51044	ribonucleoprotein complex assembly (GO:0022618)	IL6_alone_vs_EPA_IL6	0.024247546	'12/156	1.60E-05	0.001244134	RPL5;RPS17;SNRPD1;PTGES3;MRTO4;NOP2;EIF4H;SRSF5;SNRNP200;RPL6;EIF3A;RPS23
51045	ncRNA processing (GO:0034470)	IL6_alone_vs_EPA_IL6	2.086853929	'14/227	3.89E-05	0.0029182	RPL5;UTP4;RPLP1;RPLP0;RPL13A;RPL6;NOL6;RPS17;XRN2;MRTO4;SBDS;RPS11;EXOSC2;RPS23
51047	nuclear-transcribed mRNA catabolic process (GO:0000956)	IL6_alone_vs_EPA_IL6	3.144300686	'12/174	4.73E-05	0.003428063	RPL5;RPS17;CNOT7;RPLP1;CSDE1;MRTO4;RPLP0;RPL13A;PPP2R2A;RPS11;RPL6;RPS23
51049	viral translation (GO:0019081)	IL6_alone_vs_EPA_IL6	0.6266013	'4/13	5.32E-05	0.003640155	EIF3L;EIF3F;EIF3D;EIF3A
51051	glycolytic process through glucose-6-phosphate (GO:0061620)	IL6_alone_vs_EPA_IL6	1.069131663	'5/25	5.69E-05	0.003640155	TPI1;PKM;PGAM1;PGK1;GAPDH
51054	glucose catabolic process to pyruvate (GO:0061718)	IL6_alone_vs_EPA_IL6	1.333865356	'5/25	5.69E-05	0.003640155	TPI1;PKM;PGAM1;PGK1;GAPDH
51056	Golgi vesicle transport (GO:0048193)	IL6_alone_vs_EPA_IL6	2.279110028	'15/271	7.17E-05	0.004453216	ARF4;DYNC1I2;SEC23A;SEC24B;COPB1;RAB1B;ARFGAP3;ARCN1;SNX2;TFG;CAPZB;LMAN2;TMED2;ACTR10;SEC31A
51058	gene expression (GO:0010467)	IL6_alone_vs_EPA_IL6	-0.677956448	'19/411	9.33E-05	0.005631633	RANBP2;RPL5;MRPS15;SEH1L;RPLP1;RPLP0;AKR1B1;RPL13A;MRPL24;RPL6;RSL1D1;RPS17;U2AF2;HNRNPF;XRN2;NUP62;SRSF5;RPS11;RPS23
51059	establishment of protein localization to endoplasmic reticulum (GO:0072599)	IL6_alone_vs_EPA_IL6	4.337212823	'4/15	9.88E-05	0.005804633	RAB10;SPCS2;SRP68;SRP14
51061	mitochondrial ATP synthesis coupled electron transport (GO:0042775)	IL6_alone_vs_EPA_IL6	3.351851731	'8/85	0.000103222	0.005905374	NDUFS6;NDUFB5;NDUFS3;UQCRC1;MT-CO2;UQCRC2;CYC1;COX5B
51063	pyruvate metabolic process (GO:0006090)	IL6_alone_vs_EPA_IL6	1.462834203	'6/46	0.0001259	0.006991723	TPI1;PKM;GLO1;PGAM1;BSG;PGK1
51065	mitochondrial fusion (GO:0008053)	IL6_alone_vs_EPA_IL6	-0.594383517	'4/16	0.000129951	0.006991723	FIS1;AFG3L2;STOML2;CHCHD3
51069	regulation of intrinsic apoptotic signaling pathway (GO:2001242)	IL6_alone_vs_EPA_IL6	1.112359714	'6/47	0.000142269	0.007364101	FIS1;BCLAF1;RACK1;NDUFS3;PPIF;NOC2L
51070	negative regulation of interleukin-8 secretion (GO:2000483)	IL6_alone_vs_EPA_IL6	1.592602563	'3/7	0.000164849	0.008334438	ANXA1;TMSB4X;ANXA4
51072	regulation of apoptotic process (GO:0042981)	IL6_alone_vs_EPA_IL6	2.896743914	'29/815	0.000170049	0.008401971	ITGB1;ARF4;ARL6IP1;PRKDC;DIABLO;GLO1;FHL2;PARK7;PHB;HMGB1;AATF;HSP90B1;PRDX2;NCSTN;BCLAF1;NUP62;RACK1;SNCA;HSPA9;ANXA1;ANXA4;ANXA5;SOD1;DNAJA1;RSL1D1;AKT1S1;ANP32E;PPIF;ANP32B
51074	respiratory electron transport chain (GO:0022904)	IL6_alone_vs_EPA_IL6	2.765195926	'8/94	0.000209059	0.009783475	NDUFS6;NDUFB5;NDUFS3;UQCRC1;MT-CO2;UQCRC2;CYC1;COX5B
51076	peptide biosynthetic process (GO:0043043)	IL6_alone_vs_EPA_IL6	4.825991555	'11/174	0.000210025	0.009783475	RPL5;RSL1D1;RPS17;MRPS15;RPLP1;RPLP0;RPL13A;RPS11;MRPL24;RPL6;RPS23
51078	cellular macromolecule biosynthetic process (GO:0034645)	IL6_alone_vs_EPA_IL6	-0.555483991	'17/367	0.00021151	0.009783475	RPL5;MRPS15;RPLP1;RPLP0;AKR1B1;RPL13A;EEF2;MRPL24;RPL6;PTMS;EEF1B2;RSL1D1;RPS17;NUP62;MCM4;RPS11;RPS23
51079	negative regulation of programmed cell death (GO:0043069)	IL6_alone_vs_EPA_IL6	3.238617041	'18/408	0.000252002	0.011413606	HSPA9;ARF4;ANXA1;ARL6IP1;PRKDC;ANXA4;GLO1;ANXA5;FHL2;PARK7;AATF;HSP90B1;DNAJA1;PRDX2;PSMC5;NUP62;PPIF;SNCA
51081	negative regulation of cellular amide metabolic process (GO:0034249)	IL6_alone_vs_EPA_IL6	0.680150276	'7/75	0.00029455	0.013068391	CNOT7;ROCK1;RACK1;RPL13A;IGF2BP2;GAPDH;SNCA
51084	negative regulation of supramolecular fiber organization (GO:1902904)	IL6_alone_vs_EPA_IL6	1.336453427	'6/54	0.000309545	0.013195093	LIMA1;HSPA8;STMN1;TWF1;PFN1;SNCA
51085	regulation of anoikis (GO:2000209)	IL6_alone_vs_EPA_IL6	-0.07209767	'4/20	0.000327738	0.013701972	ITGB1;MYBBP1A;PTRH2;ITGA5
51087	ribosome assembly (GO:0042255)	IL6_alone_vs_EPA_IL6	2.341545297	'6/56	0.000378164	0.014971602	RPL5;RPS17;MRTO4;NOP2;SBDS;RPL6
51089	removal of superoxide radicals (GO:0019430)	IL6_alone_vs_EPA_IL6	1.592602563	'3/9	0.000385679	0.014971602	PRDX2;SOD2;SOD1
51091	mitochondrial protein processing (GO:0034982)	IL6_alone_vs_EPA_IL6	-0.96391444	'3/9	0.000385679	0.014971602	AFG3L2;STOML2;UQCRC2
51093	cellular response to superoxide (GO:0071451)	IL6_alone_vs_EPA_IL6	1.915377061	'3/9	0.000385679	0.014971602	PRDX2;SOD2;SOD1
51094	protein peptidyl-prolyl isomerization (GO:0000413)	IL6_alone_vs_EPA_IL6	0.84503795	'5/37	0.000394815	0.014971602	PPIF;CWC27;PPIL3;PPIA;FKBP3
51095	mitochondrial calcium ion transmembrane transport (GO:0006851)	IL6_alone_vs_EPA_IL6	1.007662606	'4/21	0.000399426	0.014971602	AFG3L2;STOML2;PHB;MICU1
51099	negative regulation of protein polymerization (GO:0032272)	IL6_alone_vs_EPA_IL6	3.336367907	'4/22	0.000481648	0.017165614	STMN1;TWF1;PFN1;SNCA
51101	negative regulation of actin filament polymerization (GO:0030837)	IL6_alone_vs_EPA_IL6	1.748020783	'4/22	0.000481648	0.017165614	GSN;TMSB4X;TWF1;PFN1
51103	viral life cycle (GO:0019058)	IL6_alone_vs_EPA_IL6	1.016321225	'8/107	0.000505489	0.017499985	RANBP2;SEH1L;CTBP2;RAB1B;NUP62;ITGAV;PPIA;KPNB1
51105	regulation of plasma membrane bounded cell projection assembly (GO:0120032)	IL6_alone_vs_EPA_IL6	1.333865356	'5/39	0.000507129	0.017499985	CAPZB;PODXL;RDX;FSCN1;TWF1
51112	peptidyl-proline modification (GO:0018208)	IL6_alone_vs_EPA_IL6	0.84503795	'5/40	0.000571543	0.017865344	PPIF;CWC27;PPIL3;PPIA;FKBP3
51113	positive regulation of cysteine-type endopeptidase activity involved in apoptotic process (GO:0043280)	IL6_alone_vs_EPA_IL6	0.114201474	'8/109	0.000572093	0.017865344	FIS1;DAP;GSN;DIABLO;RACK1;ANP32B;HMGB1;SNCA
51117	regulation of monooxygenase activity (GO:0032768)	IL6_alone_vs_EPA_IL6	1.007662606	'4/23	0.000575241	0.017865344	LYPLA1;SPR;DDAH1;SNCA
51118	negative regulation of intrinsic apoptotic signaling pathway (GO:2001243)	IL6_alone_vs_EPA_IL6	2.605426603	'6/62	0.00065717	0.020122363	DNAJA1;NDUFS3;PPIF;PARK7;NOC2L;SOD2
51120	ATP generation from ADP (GO:0006757)	IL6_alone_vs_EPA_IL6	1.748020783	'4/24	0.000681052	0.020482846	TPI1;PKM;PGAM1;PGK1
51122	vesicle coating (GO:0006901)	IL6_alone_vs_EPA_IL6	1.924812504	'6/63	0.000716052	0.020482846	SEC23A;SEC24B;TFG;RAB1B;TMED2;SEC31A
51124	COPII vesicle coating (GO:0048208)	IL6_alone_vs_EPA_IL6	-0.184872383	'6/63	0.000716052	0.020482846	SEC23A;SEC24B;TFG;RAB1B;TMED2;SEC31A
51128	ribosomal large subunit biogenesis (GO:0042273)	IL6_alone_vs_EPA_IL6	1.60118712	'6/63	0.000716052	0.020482846	RPL5;RSL1D1;MRTO4;NOP2;NOC2L;RPL6
51130	oxidative phosphorylation (GO:0006119)	IL6_alone_vs_EPA_IL6	0.099535674	'3/11	0.000738548	0.020584653	UQCRC1;MT-CO2;UQCRC2
51132	UDP-N-acetylglucosamine biosynthetic process (GO:0006048)	IL6_alone_vs_EPA_IL6	2.917068381	'3/11	0.000738548	0.020584653	NAGK;GNPDA1;GFPT1
51134	regulation of autophagy (GO:0010506)	IL6_alone_vs_EPA_IL6	3.237644431	'11/203	0.000775168	0.021331828	UCHL1;DAP;ROCK1;IFI16;EXOC7;USP10;ATP6V1H;HMGB1;ATP6V0D1;GAPDH;TRIM22
51142	amino sugar biosynthetic process (GO:0046349)	IL6_alone_vs_EPA_IL6	2.331260468	'3/12	0.000972295	0.02417703	NAGK;GNPDA1;GFPT1
51143	UDP-N-acetylglucosamine metabolic process (GO:0006047)	IL6_alone_vs_EPA_IL6	2.331260468	'3/12	0.000972295	0.02417703	GNPDA1;NAGK;GFPT1
51144	cellular response to arsenic-containing substance (GO:0071243)	IL6_alone_vs_EPA_IL6	2.331260468	'3/12	0.000972295	0.02417703	GSTO1;PPIF;HNRNPA1
51146	cargo loading into COPII-coated vesicle (GO:0090110)	IL6_alone_vs_EPA_IL6	2.331260468	'3/12	0.000972295	0.02417703	SEC24B;SEC23A;SEC31A
51148	cytoplasmic translational initiation (GO:0002183)	IL6_alone_vs_EPA_IL6	0.450010163	'3/12	0.000972295	0.02417703	EIF4H;EIF3D;EIF3A
51150	positive regulation of blood vessel endothelial cell migration (GO:0043536)	IL6_alone_vs_EPA_IL6	1.486717343	'5/45	0.000989768	0.02417703	STAT5A;ANXA1;TMSB4X;HMOX1;HMGB1
51152	nicotinamide nucleotide metabolic process (GO:0046496)	IL6_alone_vs_EPA_IL6	0.650689572	'5/45	0.000989768	0.02417703	TPI1;PKM;PGAM1;PGK1;GPD1L
51153	negative regulation of protein phosphorylation (GO:0001933)	IL6_alone_vs_EPA_IL6	1.8666033	'10/178	0.001012202	0.024359508	DUSP3;PRKDC;MVP;DNAJC10;AKT1S1;RACK1;BGN;PARK7;GPD1L;ENG
51155	RNA export from nucleus (GO:0006405)	IL6_alone_vs_EPA_IL6	0.812122572	'8/119	0.001019648	0.024359508	RANBP2;SEH1L;U2AF2;NUP62;XPO5;SRSF5;HNRNPA1;NOL6
51159	cargo loading into vesicle (GO:0035459)	IL6_alone_vs_EPA_IL6	0.152856507	'4/27	0.001080328	0.025254129	SEC23A;SEC24B;TMED2;SEC31A
51161	negative regulation of protein modification by small protein conjugation or removal (GO:1903321)	IL6_alone_vs_EPA_IL6	0.650689572	'5/46	0.001095118	0.025327522	DNAJA1;RPL5;UBXN1;U2AF2;PARK7
51164	antigen processing and presentation of exogenous peptide antigen via MHC class II (GO:0019886)	IL6_alone_vs_EPA_IL6	0.380412125	'7/97	0.001385302	0.031047893	DYNC1I2;SEC23A;SEC24B;CAPZB;CTSD;ACTR10;SEC31A
51166	antigen processing and presentation of exogenous peptide antigen (GO:0002478)	IL6_alone_vs_EPA_IL6	0.177668327	'7/97	0.001385302	0.031047893	DYNC1I2;SEC23A;SEC24B;CAPZB;CTSD;ACTR10;SEC31A
51167	antigen processing and presentation of peptide antigen via MHC class II (GO:0002495)	IL6_alone_vs_EPA_IL6	0.177668327	'7/98	0.001470431	0.03261956	DYNC1I2;SEC23A;SEC24B;CAPZB;CTSD;ACTR10;SEC31A
51168	negative regulation of hydrogen peroxide-induced cell death (GO:1903206)	IL6_alone_vs_EPA_IL6	2.331260468	'3/14	0.001568382	0.034096614	RACK1;TXN;PARK7
51170	mitochondrial electron transport, ubiquinol to cytochrome c (GO:0006122)	IL6_alone_vs_EPA_IL6	0.207301774	'3/14	0.001568382	0.034096614	UQCRC1;UQCRC2;CYC1
51172	protein complex assembly (GO:0006461)	IL6_alone_vs_EPA_IL6	2.090697012	'10/189	0.001588726	0.034125801	AFG3L2;STOML2;SEC23A;SEC24B;SNX2;TFG;RAB1B;AKR1B1;TMED2;SEC31A
51174	negative regulation of protein ubiquitination (GO:0031397)	IL6_alone_vs_EPA_IL6	0.84503795	'5/50	0.001601119	0.034125801	DNAJA1;RPL5;UBXN1;U2AF2;PARK7
51176	ribosomal large subunit assembly (GO:0000027)	IL6_alone_vs_EPA_IL6	0.324486822	'4/30	0.001620342	0.034200237	RPL5;NOP2;MRTO4;RPL6
51178	negative regulation of intracellular signal transduction (GO:1902532)	IL6_alone_vs_EPA_IL6	1.265426652	'9/161	0.001849811	0.03866816	DUSP3;USP10;TMSB4X;AKT1S1;PPIF;RACK1;NDUFS3;GPD1L;NOC2L
51179	apoptotic DNA fragmentation (GO:0006309)	IL6_alone_vs_EPA_IL6	1.915377061	'3/15	0.001935781	0.039330728	HMGB2;HMGB1;KPNB1
51181	regulation of establishment of cell polarity (GO:2000114)	IL6_alone_vs_EPA_IL6	1.915377061	'3/15	0.001935781	0.039330728	GSN;ROCK1;RACK1
51185	regulation of cellular response to stress (GO:0080135)	IL6_alone_vs_EPA_IL6	0.13100461	'7/104	0.002070311	0.041674604	RANBP2;HSPA8;RSL1D1;SEH1L;PTGES3;NUP62;AKT1S1
51186	regulation of cellular response to heat (GO:1900034)	IL6_alone_vs_EPA_IL6	-0.000859003	'6/78	0.002186359	0.043210887	RANBP2;HSPA8;SEH1L;PTGES3;NUP62;AKT1S1
51190	tRNA-containing ribonucleoprotein complex export from nucleus (GO:0071431)	IL6_alone_vs_EPA_IL6	0.6266013	'4/33	0.002324029	0.044711855	RANBP2;SEH1L;NUP62;NOL6
51193	positive regulation of intrinsic apoptotic signaling pathway (GO:2001244)	IL6_alone_vs_EPA_IL6	1.160514593	'4/33	0.002324029	0.044711855	FIS1;BCLAF1;RACK1;SOD1
51195	RNA transport (GO:0050658)	IL6_alone_vs_EPA_IL6	4.930737338	'6/80	0.002486297	0.047414122	RANBP2;U2AF2;XPO5;NUP62;SRSF5;HNRNPA1
51197	positive regulation of DNA binding (GO:0043388)	IL6_alone_vs_EPA_IL6	1.524780963	'4/34	0.002598857	0.049129703	HMGB2;TXN;PARK7;HMGB1
51199	cellular iron ion homeostasis (GO:0006879)	IL6_alone_vs_EPA_IL6	1.657490739	'5/56	0.002655967	0.049776492	HMOX1;ATP6V0D1;HMOX2;FTL;SOD1

Vehicle vs IL-6

	PATHWAY	COMPARISON	LOG_ODDS	OVERLAP	NOMINAL.P.value	ADJUSTED.P.value	...
88587	SRP-dependent cotranslational protein targeting to membrane, docking (GO:0006615)	vehicle_vs_IL6_alone	0.47531495	'28/88	2.62E-24	1.40E-20	RPL4;RPL5;RPL31;RPL34;RPLP1;RPL8;RPL6;RPL7;RPS4X;RPL7A;RPS17;RPS3;RPS11;RPS13;RPS7;RPL23;RPS6;RPL13A;RPS3A;SRP68;RPS25;RPL27A;RPL37A;RPL24;RPL29;RPL28;RPS21;RPS23
88588	SRP-dependent cotranslational protein targeting to membrane (GO:0006614)	vehicle_vs_IL6_alone	0.46920393	'29/100	7.80E-24	1.41E-20	[illegible]
88590	translational elongation (GO:0006414)	vehicle_vs_IL6_alone	0.49003797	'35/162	8.74E-24	1.41E-20	[illegible]
88592	SRP-dependent cotranslational protein targeting to membrane, translocation (GO:0006616)	vehicle_vs_IL6_alone	0.47531495	'28/92	1.05E-23	1.41E-20	[illegible]
88594	nuclear-transcribed mRNA catabolic process, nonsense-mediated decay (GO:0000184)	vehicle_vs_IL6_alone	0.40447628	'31/125	3.97E-23	4.25E-20	[illegible]
88596	SRP-dependent cotranslational protein targeting to membrane, signal sequence processing (GO:0006618)	vehicle_vs_IL6_alone	0.44970398	'29/108	8.85E-23	5.26E-20	[illegible]
88597	DNA-templated viral transcription (GO:0039695)	vehicle_vs_IL6_alone	0.43044063	'29/108	8.85E-23	5.26E-20	[illegible]
88598	middle viral transcription (GO:0019084)	vehicle_vs_IL6_alone	0.43044063	'29/108	8.85E-23	5.26E-20	[illegible]
88599	RNA-templated viral transcription (GO:0039696)	vehicle_vs_IL6_alone	0.43044063	'29/108	8.85E-23	5.26E-20	[illegible]
88600	early viral transcription (GO:0019085)	vehicle_vs_IL6_alone	0.43044063	'29/110	1.57E-22	8.39E-20	[illegible]
88601	nuclear-transcribed mRNA catabolic process, 5'-3' exonucleolytic nonsense-mediated decay (GO:0070479)	vehicle_vs_IL6_alone	0.39260011	'29/111	2.08E-22	1.01E-19	[illegible]
88602	SRP-dependent cotranslational protein targeting to membrane, signal sequence recognition (GO:0006617)	vehicle_vs_IL6_alone	0.45506697	'28/102	2.52E-22	1.12E-19	[illegible]
88603	late viral transcription (GO:0019086)	vehicle_vs_IL6_alone	0.43044063	'29/112	2.74E-22	1.13E-19	[illegible]
88604	tRNA aminoacylation for protein translation (GO:0006418)	vehicle_vs_IL6_alone	0.50669721	'35/179	2.98E-22	1.14E-19	[illegible]
88606	translational termination (GO:0006415)	vehicle_vs_IL6_alone	0.46627116	'33/159	6.63E-22	2.36E-19	[illegible]
88608	viral RNA editing (GO:0075527)	vehicle_vs_IL6_alone	0.37401166	'29/119	1.76E-21	5.56E-19	[illegible]
88609	viral transcription (GO:0019083)	vehicle_vs_IL6_alone	0.37401166	'29/119	1.76E-21	5.56E-19	[illegible]
88611	nuclear-transcribed mRNA catabolic process, 3'-5' exonucleolytic nonsense-mediated decay (GO:0070478)	vehicle_vs_IL6_alone	0.30177237	'30/143	3.66E-20	1.09E-17	[illegible]
88612	regulation of translation by machinery localization (GO:0043143)	vehicle_vs_IL6_alone	0.39595497	'34/194	4.68E-20	1.32E-17	[illegible]
88613	cleavage involved in rRNA processing (GO:0000469)	vehicle_vs_IL6_alone	0.44907907	'33/184	8.01E-20	2.14E-17	[illegible]
88614	maturation of LSU-rRNA (GO:0000470)	vehicle_vs_IL6_alone	0.41211531	'34/203	2.08E-19	5.30E-17	[illegible]
88616	plastid translation (GO:0032544)	vehicle_vs_IL6_alone	0.37167269	'30/152	2.31E-19	5.38E-17	[illegible]
88617	maintenance of translational fidelity (GO:1990145)	vehicle_vs_IL6_alone	0.37167269	'30/152	2.31E-19	5.38E-17	[illegible]
88618	N-terminal peptidyl-proline dimethylation involved in translation (GO:0071891)	vehicle_vs_IL6_alone	0.37167269	'30/153	2.81E-19	6.28E-17	[illegible]
88619	chloroplast rRNA processing (GO:1901259)	vehicle_vs_IL6_alone	0.43597976	'32/181	4.60E-19	8.21E-17	[illegible]
88620	rRNA 5'-end processing (GO:0000967)	vehicle_vs_IL6_alone	0.43597976	'32/181	4.60E-19	8.21E-17	[illegible]
88621	rRNA 3'-end processing (GO:0031125)	vehicle_vs_IL6_alone	0.43597976	'32/181	4.60E-19	8.21E-17	[illegible]
88623	maturation of 4.5S rRNA (GO:0000476)	vehicle_vs_IL6_alone	0.43597976	'32/181	4.60E-19	8.21E-17	[illegible]
88624	snoRNA release from pre-rRNA (GO:1990417)	vehicle_vs_IL6_alone	0.43597976	'32/181	4.60E-19	8.21E-17	[illegible]
88625	maturation of 2S rRNA (GO:0000475)	vehicle_vs_IL6_alone	0.43597976	'32/181	4.60E-19	8.21E-17	[illegible]
88626	maturation of 5S rRNA (GO:0000481)	vehicle_vs_IL6_alone	0.41860191	'32/182	5.47E-19	9.44E-17	[illegible]
88627	cytoplasmic translation (GO:0002181)	vehicle_vs_IL6_alone	0.35391024	'30/157	6.08E-19	1.02E-16	[illegible]
88629	maturation of SSU-rRNA (GO:0030490)	vehicle_vs_IL6_alone	0.43597976	'32/186	1.07E-18	1.74E-16	[illegible]
88631	rRNA modification (GO:0000154)	vehicle_vs_IL6_alone	0.41860191	'32/187	1.27E-18	1.94E-16	[illegible]
88633	maturation of 5.8S rRNA (GO:0000460)	vehicle_vs_IL6_alone	0.40140982	'32/187	1.27E-18	1.94E-16	[illegible]
88635	rRNA processing (GO:0006364)	vehicle_vs_IL6_alone	0.43597976	'32/188	1.49E-18	2.22E-16	[illegible]
88637	mitochondrial translation (GO:0032543)	vehicle_vs_IL6_alone	0.33634232	'30/167	3.77E-18	5.45E-16	[illegible]
[illegible]	[illegible]	[illegible]	[illegible]	[illegible]	[illegible]	[illegible]	[illegible]

95960	negative regulation of macromolecule metabolic process (GO:0010605)	vehicle_vs_IL6_alone	0.93216643	'12/124	3.87E-05	0.001904758	[illegible]
95961	DNA-templated transcription, termination (GO:0006353)	vehicle_vs_IL6_alone	2.53334298	'11/106	4.25E-05	0.002051386	CPSF7;CPSF1;CAVIN1;CCNH;SRSF2;SRSF3;SYMPK;SNRPD3;SRSF6;SRSF7;SNRPB
95963	regulation of telomerase RNA localization to Cajal body (GO:1904872)	vehicle_vs_IL6_alone	0.47904411	'5/18	4.81E-05	0.002237673	[illegible]
95970	regulation of cellular ketone metabolic process (GO:0010565)	vehicle_vs_IL6_alone	-0.4782468	'8/61	9.05E-05	0.00394516	PSMB7;NQO1;PSMC5;PSMD11;PSMC6;CAV1;PSME1;PSMD1
95971	regulation of nitric oxide biosynthetic process (GO:0045428)	vehicle_vs_IL6_alone	1.27907779	'6/32	9.11E-05	0.00394516	HSP90AB1;DDAH2;KHSRP;CAV1;CLU;CD34
95972	positive regulation of nucleocytoplasmic transport (GO:0046824)	vehicle_vs_IL6_alone	2.86912319	'6/33	0.000109127	0.00464624	KHDRBS1;CPSF6;BAG3;NEDD4;TPR;IPO5
95974	ribosomal large subunit biogenesis (GO:0042273)	vehicle_vs_IL6_alone	1.54745514	'8/63	0.00011419	0.004780739	RPL5;RPL7A;RSL1D1;MRTO4;NHP2;RPL24;RPL6;RPL7
95976	mRNA-containing ribonucleoprotein complex export from nucleus (GO:0071427)	vehicle_vs_IL6_alone	0.2253904	'10/100	0.000128784	0.005262566	SEH1L;CPSF1;TPR;HNRNPA2B1;SRSF2;SRSF3;SYMPK;SRSF6;SRSF7;EIF4E
95977	apoptotic mitochondrial changes (GO:0008637)	vehicle_vs_IL6_alone	-0.7844105	'6/34	0.000129888	0.005262566	FIS1;BID;DNM1L;SOD2;CLU;HSPD1
95979	negative regulation of protein polymerization (GO:0032272)	vehicle_vs_IL6_alone	3.18881072	'5/22	0.000136731	0.005284109	HIP1R;TWF1;MAPRE1;TUBB4A;PFN2
95987	protein modification process (GO:0036211)	vehicle_vs_IL6_alone	0.71196952	'9/84	0.000164152	0.006154457	DUSP12;PARP4;UBE2I;YES1;PRKDC;RPN1;MAN2B1;PLOD1;LOXL2
95988	regulation of cellular response to stress (GO:0080135)	vehicle_vs_IL6_alone	0.17872669	'10/104	0.000178568	0.006452719	[illegible]
95989	mRNA transport (GO:0051028)	vehicle_vs_IL6_alone	4.20430706	'10/104	0.000178568	0.006452719	SEH1L;CPSF1;TPR;HNRNPA2B1;SRSF2;SRSF3;SYMPK;SRSF6;SRSF7;EIF4E
95993	regulation of cellular amine metabolic process (GO:0033238)	vehicle_vs_IL6_alone	-0.6366011	'7/51	0.000186361	0.006501938	PSMB7;NQO1;PSMC5;PSMD11;PSMC6;PSME1;PSMD1
95996	negative regulation of cellular process (GO:0048523)	vehicle_vs_IL6_alone	0.37936916	'27/534	0.000192115	0.006560832	APP;COL18A1;NDRG3;SNX12;AKR1B1;CLU;PPP2CA;PODXL;ARHGDIA;LMNA;ARHGDIB;TMEM109;CD34;HRAS;CAV1;P3H1;TSC2;MIF;SOD2;NME1;CNOT7;CDH13;MYH9;SRSF6;EEF1E1;HSPA1B;VAMP3
96001	chaperone mediated protein folding requiring cofactor (GO:0051085)	vehicle_vs_IL6_alone	3.18881072	'5/24	0.000212244	0.007015229	HSPA8;HSPA5;HYOU1;HSPA2;HSPA1B
96003	regulation of apoptotic process (GO:0042981)	vehicle_vs_IL6_alone	2.7272173	'36/815	0.000258162	0.00842212	ANP32A;PRKDC;DIABLO;GSTP1;GLO1;AATF;CLU;HSPD1;NCSTN;BAG3;ARHGDIA;CFL1;RPS3;BID;DNM1L;TXNDC5;ANXA1;MAP2K2;HSPA5;STAT3;RPS6;HIP1R;TNFRSF10B;RPS3A;MIF;ATAD3A;YWHAZ;NME1;RSL1D1;DDAH2;APEX1;AKT1S1;EEF1E1;TARDBP;HSPA1B;TPT1
96005	negative regulation of supramolecular fiber organization (GO:1902904)	vehicle_vs_IL6_alone	1.08697713	'7/54	0.000268225	0.008638235	HSPA8;HIP1R;TWF1;MAPRE1;CLU;TUBB4A;PFN2
96006	protein tetramerization (GO:0051262)	vehicle_vs_IL6_alone	0.78682402	'9/90	0.000278006	0.008839878	HMGCL;CPSF7;CPSF6;HPRT1;DNM1L;ALDOA;SOD2;FARSA;S100A10
96008	negative regulation of programmed cell death (GO:0043069)	vehicle_vs_IL6_alone	3.05099004	'22/408	0.000311024	0.009766158	ANXA1;HSPA5;PRKDC;GSTP1;GLO1;STAT3;RPS3A;MIF;AATF;ATAD3A;YBX3;YWHAZ;HSPD1;PSMC5;BAG3;DDAH2;ARHGDIA;CFL1;BID;HSPA1B;TPT1;TXNDC5
96012	regulation of RNA export from nucleus (GO:0046831)	vehicle_vs_IL6_alone	1.27545611	'4/15	0.000342697	0.010498222	KHDRBS1;CPSF6;TPR;IWS1
96015	respiratory electron transport chain (GO:0022904)	vehicle_vs_IL6_alone	2.46084423	'9/94	0.000385348	0.01135119	NDUFB9;NDUFA6;NDUFB6;NDUFS6;COX4I1;NDUFS2;COX7C;COX5A;NDUFV1
96017	regulation of actin cytoskeleton organization (GO:0032956)	vehicle_vs_IL6_alone	1.05862773	'8/75	0.000388616	0.01135119	CDC42;ROCK1;ARHGDIA;ARHGDIB;RDX;HIP1R;TWF1;PHPT1
96021	negative regulation of nitric oxide metabolic process (GO:1904406)	vehicle_vs_IL6_alone	2.4456929	'3/7	0.000428561	0.012233482	KHSRP;CAV1;CD34
96025	antigen processing and presentation of exogenous peptide antigen via MHC class I (GO:0042590)	vehicle_vs_IL6_alone	1.40910222	'8/78	0.00050783	0.014333369	PSMB7;PSMC5;PSMD11;PSMC6;SNAP23;PSME1;PSMD1;VAMP3
96027	cortical cytoskeleton organization (GO:0030865)	vehicle_vs_IL6_alone	0.7217525	'5/29	0.000538006	0.015016342	CDC42;ROCK1;EPB41L3;RAB13;EZR
96029	RNA 3'-end processing (GO:0031123)	vehicle_vs_IL6_alone	0.58021979	'7/61	0.000573331	0.015544882	CPSF7;CPSF1;SRSF2;SRSF3;SYMPK;SRSF6;SRSF7
96031	positive regulation of signal transduction by p53 class mediator (GO:1901798)	vehicle_vs_IL6_alone	-0.4734186	'4/17	0.000575507	0.015544882	RPS7;RPL23;EEF1E1;CDK5RAP3
96033	regulation of early endosome to late endosome transport (GO:2000641)	vehicle_vs_IL6_alone	1.53933741	'4/17	0.000575507	0.015544882	MAP2K2;RDX;SNX12;EZR
96035	RNA transport (GO:0050658)	vehicle_vs_IL6_alone	4.87700535	'8/80	0.000602599	0.016103507	[illegible]
96037	regulation of actin cytoskeleton reorganization (GO:2000249)	vehicle_vs_IL6_alone	0.86010542	'5/30	0.000633183	0.016234411	CDC42;ARHGDIA;ARHGDIB;PHPT1;HRAS
96039	'de novo' posttranslational protein folding (GO:0051084)	vehicle_vs_IL6_alone	0.59537172	'5/30	0.000633183	0.016234411	HSPA8;HSPA5;HYOU1;HSPA2;HSPA1B
96040	negative regulation of G2/M transition of mitotic cell cycle (GO:0010972)	vehicle_vs_IL6_alone	-0.6739911	'7/62	0.000633349	0.016234411	PSMB7;PSMC5;PSMD11;PSMC6;PSME1;PSMD1;CDK5RAP3
96044	positive regulation of mitochondrial calcium ion concentration (GO:0051561)	vehicle_vs_IL6_alone	0.63410595	'3/8	0.000673669	0.016590762	BCAP31;FIS1;MCU
96046	regulation of amyloid precursor protein catabolic process (GO:1902991)	vehicle_vs_IL6_alone	1.85988499	'3/8	0.000673669	0.016590762	TMED10;ROCK1;FLOT2
96047	myelin maintenance (GO:0043217)	vehicle_vs_IL6_alone	1.44400158	'3/8	0.000673669	0.016590762	EPB41L3;AHNAK2;CLU
96049	angiogenesis involved in wound healing (GO:0060055)	vehicle_vs_IL6_alone	1.85988499	'3/8	0.000673669	0.016590762	MCAM;PRCP;CD34
96053	regulation of amyloid-beta formation (GO:1902003)	vehicle_vs_IL6_alone	1.86211191	'4/18	0.000726195	0.017540402	APP;TMED10;ROCK1;CLU
96058	negative regulation of cellular biosynthetic process (GO:0031327)	vehicle_vs_IL6_alone	1.1262258	'6/47	0.000800251	0.018731506	ACADVL;KHSRP;CAV1;PGP;ERLIN2;CD34
96059	regulated exocytosis (GO:0045055)	vehicle_vs_IL6_alone	4.34492992	'11/148	0.000805335	0.018731506	APP;SPARC;TMED10;VWF;CD109;STXBP2;ENDOD1;TAGLN2;ALDOA;CLU;MANF
96063	protein targeting to lysosome (GO:0006622)	vehicle_vs_IL6_alone	2.27799532	'4/19	0.000902776	0.020430399	HSPA8;NEDD4;CLU;RAB7A
96065	protein heterotetramerization (GO:0051290)	vehicle_vs_IL6_alone	0.15403662	'4/19	0.000902776	0.020430399	CPSF7;CPSF6;FARSA;S100A10
96067	ATP synthesis coupled electron transport (GO:0042773)	vehicle_vs_IL6_alone	0.44061298	'3/9	0.000992798	0.022267036	NDUFB6;NDUFS2;NDUFV1
96069	positive regulation of protein ubiquitination involved in ubiquitin-dependent protein catabolic process (GO:2000060)	vehicle_vs_IL6_alone	1.87108052	'8/87	0.001052468	0.023327148	PSMB7;PSMC5;PSMD11;PSMC6;PSME1;PSMD1;ANAPC4;CLU
96071	protein localization to plasma membrane (GO:0072659)	vehicle_vs_IL6_alone	2.19922714	'10/130	0.001058637	0.023327148	ROCK1;RILPL1;EPB41L3;RDX;RAB13;FLOT2;EZR;OPTN;SPTBN1;S100A10
96073	negative regulation of protein phosphorylation (GO:0001933)	vehicle_vs_IL6_alone	1.6560542	'12/178	0.001118916	0.024441011	PRKDC;CAV1;GSTP1;MVP;CHORDC1;AKT1S1;BGN;TSC2;STRAP;TARDBP;CDK5RAP3;ENG
96075	regulation of wound healing (GO:0061041)	vehicle_vs_IL6_alone	1.37722378	'5/34	0.00114402	0.024773954	ANXA1;CAV1;CD109;SRSF6;HRAS
96076	positive regulation of cell motility (GO:2000147)	vehicle_vs_IL6_alone	2.46850699	'12/179	0.001174296	0.025212241	SUN2;HSPA5;CAVIN1;PODXL;RDX;CDH13;LAMB1;PHPT1;MAPRE1;SOD2;HRAS;CCAR1
96078	endothelial cell migration (GO:0043542)	vehicle_vs_IL6_alone	1.8643449	'5/35	0.001309081	0.027867896	PXN;RAB13;CDH13;MYH9;LOXL2
96082	regulation of ruffle assembly (GO:1900027)	vehicle_vs_IL6_alone	1.53933741	'4/21	0.001342795	0.028109174	CAV1;RDX;HRAS;PFN2
96083	mitotic nuclear envelope reassembly (GO:0007084)	vehicle_vs_IL6_alone	0.63410595	'3/10	0.00139346	0.028691566	PPP2CA;LMNA;BANF1
96089	nuclear import (GO:0051170)	vehicle_vs_IL6_alone	0.97064951	'7/71	0.001427901	0.028926513	TPR;STAT3;TSC2;GEMIN5;SNRPD3;KPNB1;SNRPB
96091	nuclear export (GO:0051168)	vehicle_vs_IL6_alone	1.87108052	'8/92	0.0015136	0.030417305	CHORDC1;XPO5;TPR;SRSF2;SRSF3;SRSF6;SRSF7;EIF4E
96093	regulation of ubiquitin-protein ligase activity involved in mitotic cell cycle (GO:0051439)	vehicle_vs_IL6_alone	3.68041612	'7/72	0.001550097	0.030903524	PSMB7;PSMC5;PSMD11;PSMC6;PSME1;PSMD1;ANAPC4
96097	membrane fusion (GO:0061025)	vehicle_vs_IL6_alone	1.1262258	'6/54	0.001675204	0.032875881	FIS1;CHCHD3;SNAP23;CHORDC1;DNM1L;VAMP3
96099	negative regulation of gene expression (GO:0010629)	vehicle_vs_IL6_alone	2.21480024	'27/618	0.001746966	0.033857614	KHDRBS1;APP;SNX12;LOXL2;IFI16;RPS3;FLOT2;IGF2BP2;CD34;HRAS;RPS13;HSPA8;MAP2K2;UBE2I;CBX3;NONO;RPL13A;CNPY2;MIF;DAP;SFPQ;CNOT7;PSPC1;COPS2;TARDBP;HSPA1B;ENG
96101	regulation of macroautophagy (GO:0016241)	vehicle_vs_IL6_alone	0.16820853	'9/116	0.001752185	0.033857614	UCHL1;EXOC7;NEDD4;ATP6V1B2;TSC2;ATP6V1H;CAPN1;PRKACA;PAFAH1B2
96103	protein localization to cell periphery (GO:1990778)	vehicle_vs_IL6_alone	5.05003627	'9/117	0.001859459	0.035290091	ROCK1;CAV1;EPB41L3;RDX;RAB13;FLOT2;EZR;SPTBN1;S100A10
96105	positive regulation of protein localization to chromosome, telomeric region (GO:1904816)	vehicle_vs_IL6_alone	0.26983958	'3/11	0.001882513	0.035290091	DKC1;TCP1;GNL3
96108	negative regulation of amyloid precursor protein catabolic process (GO:1902992)	vehicle_vs_IL6_alone	1.85988499	'3/11	0.001882513	0.035290091	ROCK1;FLOT2;CLU
96109	ribosome assembly (GO:0042255)	vehicle_vs_IL6_alone	1.86658398	'6/56	0.002024196	0.037665037	RPL5;RPS17;MRTO4;RPL24;RPL6;EIF2A
96111	positive regulation of apoptotic signaling pathway (GO:2001235)	vehicle_vs_IL6_alone	-0.4770304	'7/76	0.002122327	0.038914494	[illegible]
96115	regulation of plasma membrane bounded cell projection assembly (GO:0120032)	vehicle_vs_IL6_alone	0.86010542	'5/39	0.002148871	0.039115687	CDC42;PODXL;CAV1;RDX;TWF1
96116	negative regulation of cytoskeleton organization (GO:0051494)	vehicle_vs_IL6_alone	1.27545611	'4/24	0.002254184	0.040446496	HIP1R;TWF1;FKBP4;PFN2
96118	regulation of actin filament bundle assembly (GO:0032231)	vehicle_vs_IL6_alone	0.68794992	'4/24	0.002254184	0.040446496	CDC42;ROCK1;RDX;PFN2
96120	tRNA aminoacylation (GO:0043039)	vehicle_vs_IL6_alone	1.37722378	'5/40	0.002408876	0.042143339	AIMP2;PPA2;IARS2;FARSA;EEF1E1
96121	positive regulation of establishment of protein localization (GO:1904951)	vehicle_vs_IL6_alone	0.26983958	'3/12	0.002466191	0.042143339	DKC1;TCP1;CHORDC1
96128	purine nucleoside monophosphate biosynthetic process (GO:0009127)	vehicle_vs_IL6_alone	0.85734577	'3/12	0.002466191	0.042143339	PAICS;PFAS;GART
96131	regulation of protein ubiquitination involved in ubiquitin-dependent protein catabolic process (GO:2000058)	vehicle_vs_IL6_alone	2.27799532	'4/25	0.002633995	0.044072259	RPL5;RPS7;RPL23;CLU
96135	regulation of extrinsic apoptotic signaling pathway (GO:2001236)	vehicle_vs_IL6_alone	0.7451645	'6/59	0.002647218	0.044072259	LGALS3;GSTP1;CAV1;AKR1B1;TNFRSF10B;BID
96136	anaphase-promoting complex-dependent catabolic process (GO:0031145)	vehicle_vs_IL6_alone	-1.1997233	'7/79	0.002649248	0.044072259	PSMB7;PSMC5;PSMD11;PSMC6;PSME1;PSMD1;ANAPC4
96140	regulation of mRNA splicing, via spliceosome (GO:0048024)	vehicle_vs_IL6_alone	0.86149211	'6/60	0.002883807	0.047347214	DDX17;THRAP3;SRSF3;SRSF6;SRSF7;PRDX6
96142	regulation of G2/M transition of mitotic cell cycle (GO:0010389)	vehicle_vs_IL6_alone	4.20430706	'10/149	0.00292226	0.047666994	PSMB7;PSMC5;PSMD11;PSMC6;TUBB;PSME1;PSMD1;PRKACA;MAPRE1;TUBB4A
96144	positive regulation of protein localization to nucleus (GO:1900182)	vehicle_vs_IL6_alone	2.60300281	'5/42	0.002996285	0.048559155	[illegible]
96146	regulation of T cell receptor signaling pathway (GO:0050856)	vehicle_vs_IL6_alone	1.27545611	'4/26	0.003055464	0.049149935	LGALS3;RPS3;PHPT1;EZR
96147	positive regulation of cellular amide metabolic process (GO:0034250)	vehicle_vs_IL6_alone	-0.0864894	'6/61	0.00313586	0.049149935	RPS4X;APP;RPL5;EEF2;CLU;EIF3D
96148	regulation of cell aging (GO:0090342)	vehicle_vs_IL6_alone	1.85988499	'3/13	0.003150135	0.049149935	RSL1D1;LMNA;MIF
96151	negative regulation of reactive oxygen species biosynthetic process (GO:1903427)	vehicle_vs_IL6_alone	1.44400158	'3/13	0.003150135	0.049149935	CAV1;KHSRP;CD34
96153	negative regulation of ubiquitin-protein transferase activity (GO:0051444)	vehicle_vs_IL6_alone	-0.1477461	'3/13	0.003150135	0.049149935	RPL5;RPS7;RPL23

Table S3

Fatty Acid		Treatment		
		Control (mg/g protein)	IL-6 (mg/g protein)	EPA + IL-6 (mg/g protein)
14:0	Myristic	7.63 ± 0.37	7.68 ± 0.38	8.65 ± 0.14
16:0	Palmitic	60.09 ± 2.86	59.62 ± 3.30	66.02 ± 0.72
trans-16:1n7	Palmitelaidic	1.13 ± 0.06	1.07 ± 0.13	1.00 ± 0.03
cis-16:1n7	Palmitoleic	6.33 ± 0.54	6.61 ± 0.21	6.71 ± 0.17
18:0	Stearic	40.80 ± 2.42	39.98 ± 2.43	43.87 ± 0.44
trans-18:1n9	Elaidic	0.95 ± 0.12	1.10 ± 0.13	0.93 ± 0.08
cis-18:1n9	Oleic	84.28 ± 4.76	82.53 ± 4.13	78.91 ± 2.23
trans-18:2n6	Linoelaidic	2.36 ± 0.08	2.47 ± 0.10	2.55 ± 0.07
cis-18:2n6	Linoleic	4.39 ± 0.32	4.69 ± 0.08	5.05 ± 0.08
18:3n3	α-Linolenic	0.03 ± 0.01	0.11 ± 0.04	0.07 ± 0.02
18:3n6	γ-Linolenic	0.11 ± 0.02	0.20 ± 0.05	0.35 ± 0.002* ‡
20:0	Arachidic	0.93 ± 0.04	1.09 ± 0.08	1.00 ± 0.04
20:1n9	Eicosenoic	1.54 ± 0.11	1.41 ± 0.19	0.91 ± 0.03*
20:2n6	Eicosadienoic	0.39 ± 0.04	0.36 ± 0.01	0.51 ± 0.05
20:3n6	Dihomo-γ-linolenic	4.77 ± 0.46	4.90 ± 0.10	5.29 ± 0.20
20:4n6	Arachidonic (AA)	17.61 ± 2.13	17.85 ± 0.41	17.23 ± 0.86
20:5n3	Eicosapentaenoic (EPA)	1.24 ± 0.14	1.28 ± 0.05	26.71 ± 1.28* ‡
22:0	Behenic	1.14 ± 0.05	1.14 ± 0.11	1.19 ± 0.17
22:4n6	Docosatetraenoic	2.93 ± 0.28	3.11 ± 0.20	3.54 ± 0.14
22:5n6	Docosapentaenoic ω6	0.59 ± 0.06	0.64 ± 0.04	0.52 ± 0.04
22:5n3	Docosapentaenoic ω3	5.86 ± 0.69	6.17 ± 0.36	29.42 ± 1.91* ‡
22:6n3	Docosahexaenoic	7.05 ± 0.86	7.41 ± 0.43	8.03 ± 0.52
24:0	Lignoceric	1.94 ± 0.08	2.13 ± 0.03	2.10 ± 0.08
24:1n9	Nervonic	1.28 ± 0.03	1.28 ± 0.20	1.16 ± 0.07
Eicosapentaenoic/Arachidonic Acid Ratio (EPA/AA)		0.07 ± 0.01	0.07 ± 0.003	1.55 ± 0.11* ‡

Summary of Fatty Acid Levels in HUVECs Following Treatment with IL-6 ± EPA. Results shown as mean ± SEM. Statistical analysis were carried out by one-way ANOVA followed by *post hoc* Tukey-Kramer multiple comparisons test between groups. **p*<0.05 vs control; ‡p<0.05 vs IL-6.

Figure S1

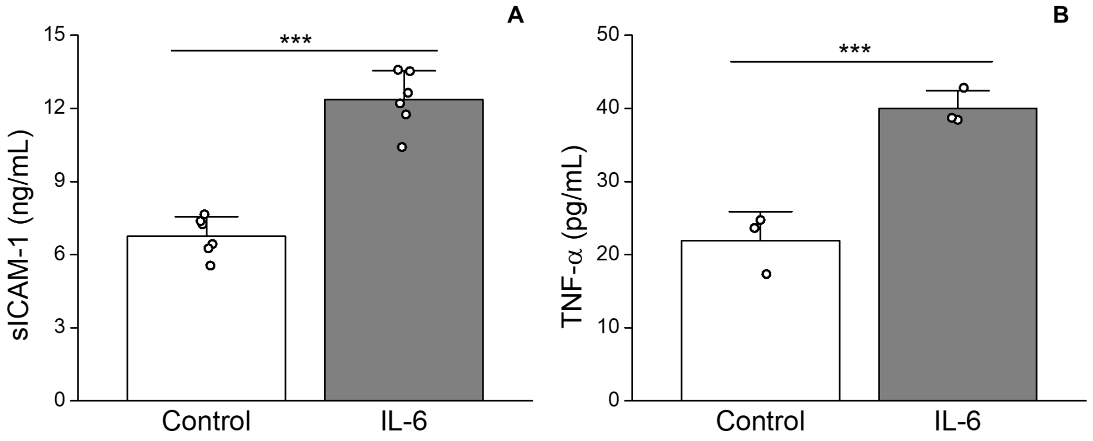

Release of (A) sICAM-1 and (B) TNF-α from HUVECs Challenged with IL-6. sICAM statistical indicators: ***$p<0.0001$ versus control (Unpaired, two-tailed Student's T-test, $t = 9.529$, $df = 10$). TNF-α statistical indicators: ***$p = 0.0026$ versus control (Unpaired, two-tailed Student's t-test, $t = 6.682$, $df = 4$). Values are mean ± SD (N = 3-6).

Figure S2

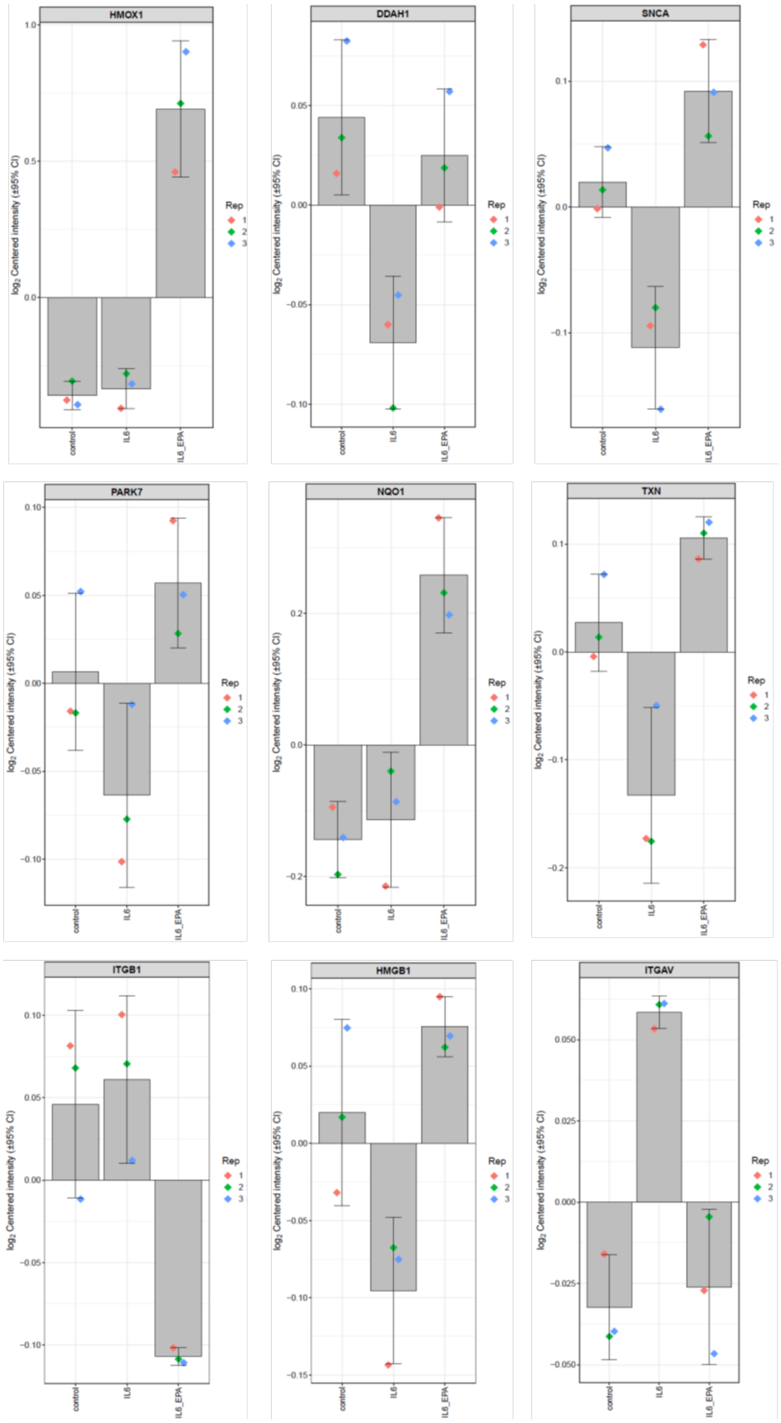

Summary of Anti-inflammatory and Antioxidant Response Proteins Modulated by EPA at 40 µM in IL-6-Challenged ECs. Key: DDAH1, dimethylarginine dimethylaminohydrolase-1; HMGB1, high mobility group box protein 1; HMOX1, heme oxygenase-1; ITGAV, integrin αV; ITGB1, integrin B1; NQO1, NAD(P)H quinone oxidoreductase-1; PARK1, Parkinson disease protein 7; SNCA; α-synuclein; TXN, thioredoxin.

Figure S3

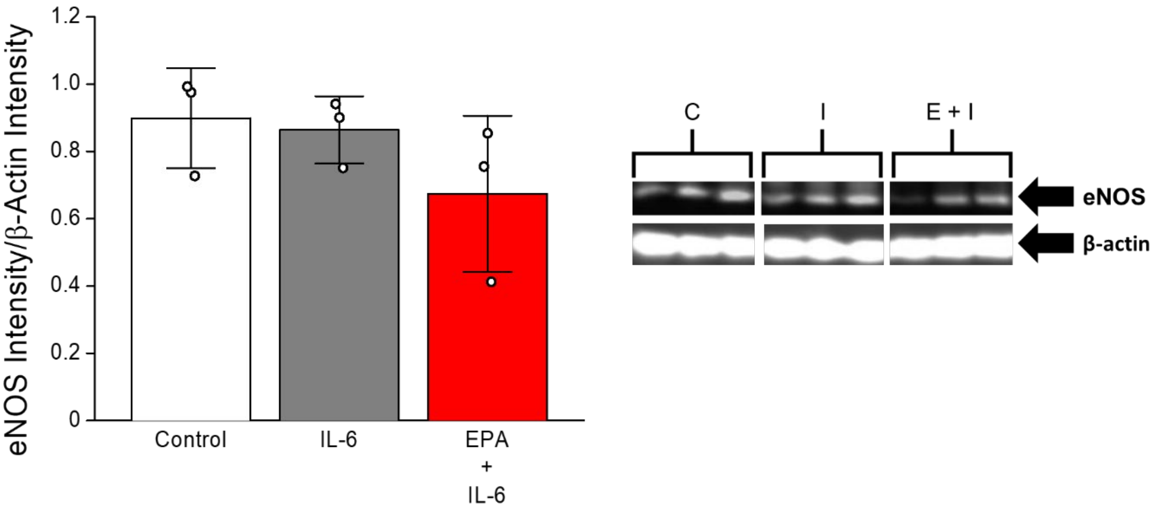

Expression of eNOS Across Treatment Groups. The expression of eNOS was normalized to β-actin levels. (Tukey-Kramer Multiple Comparisons Test; overall ANOVA: $p=0.2897$, $F = 1.534$). Values are mean ± SD ($N = 3$). Abbreviations: C, control; I, IL-6; E + I, EPA + IL-6.

Figure S4

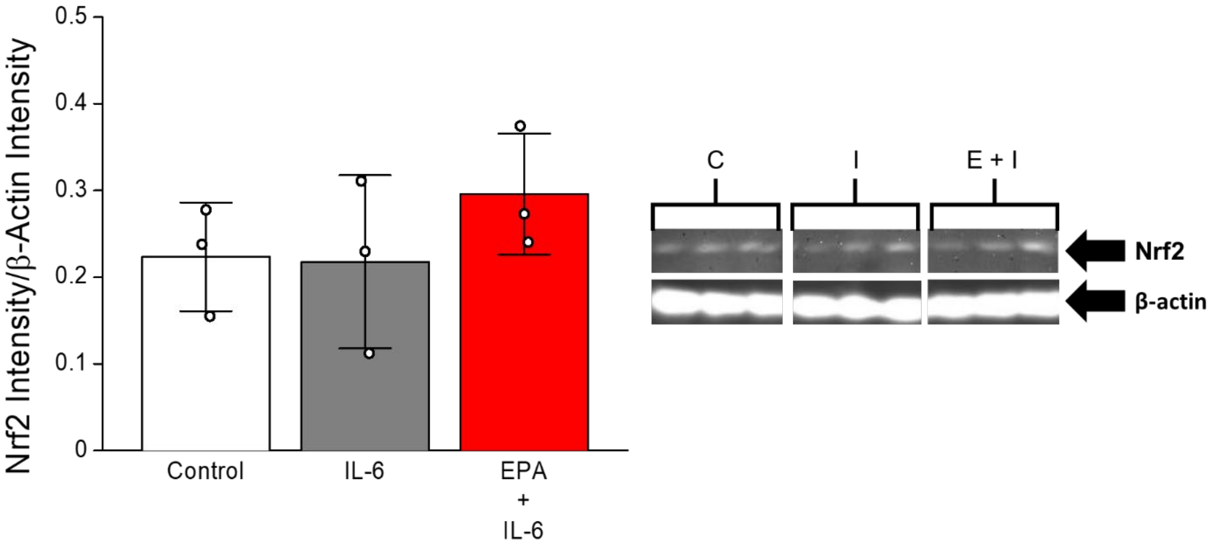

Expression of Nrf2 Across Treatment Groups. The expression of Nrf2 was normalized to β-actin levels. (Tukey-Kramer Multiple Comparisons Test; overall ANOVA: $p=0.4506$, $F = 0.9132$). Values are mean ± SD ($N = 3$). Abbreviations: C, control; I, IL-6; E + I, EPA + IL-6.
